# A mitochondrion-targeted cyanine agent for NIR-II fluorescence-guided surgery combined with intraoperative photothermal therapy to reduce prostate cancer recurrence

**DOI:** 10.1186/s12951-024-02477-6

**Published:** 2024-05-03

**Authors:** Chenchen Liu, Zong Chang, Kailei Chen, Qiang Xue, Bingxin Shu, Zhihao Wei, Xuan Zhou, Like Guo, Yuling Zhang, Yingying Pan, Qi Cao, Huageng Liang, Qinchao Sun, Xiaoping Zhang

**Affiliations:** 1 Department of Urology, Union Hospital, Tongji Medical College, Huazhong University of Science and Technology, Wuhan, 430022 China; 2 Institute of Urology, Union Hospital, Tongji Medical College, Huazhong University of Science and Technology, Wuhan, 430022 China; 3 Guangdong Provincial Key Laboratory of Biomedical Optical Imaging Technology & Center for Biomedical Optics and Molecular Imaging, Shenzhen Institute of Advanced Technology, Chinese Academy of Science, Shenzhen, 518055 China

**Keywords:** Prostate cancer, Intraoperative intervention, Fluorescence-guided surgery, Photothermal therapy, Recurrence

## Abstract

Poorly identified tumor boundaries and nontargeted therapies lead to the high recurrence rates and poor quality of life of prostate cancer patients. Near-infrared-II (NIR-II) fluorescence imaging provides certain advantages, including high resolution and the sensitive detection of tumor boundaries. Herein, a cyanine agent (CY7-4) with significantly greater tumor affinity and blood circulation time than indocyanine green was screened. By binding albumin, the absorbance of CY7-4 in an aqueous solution showed no effects from aggregation, with a peak absorbance at 830 nm and a strong fluorescence emission tail beyond 1000 nm. Due to its extended circulation time (half-life of 2.5 h) and high affinity for tumor cells, this fluorophore was used for primary and metastatic tumor diagnosis and continuous monitoring. Moreover, a high tumor signal-to-noise ratio (up to ~ 10) and excellent preferential mitochondrial accumulation ensured the efficacy of this molecule for photothermal therapy. Therefore, we integrated NIR-II fluorescence-guided surgery and intraoperative photothermal therapy to overcome the shortcomings of a single treatment modality. A significant reduction in recurrence and an improved survival rate were observed, indicating that the concept of intraoperative combination therapy has potential for the precise clinical treatment of prostate cancer.

**Graphical Abstract:**

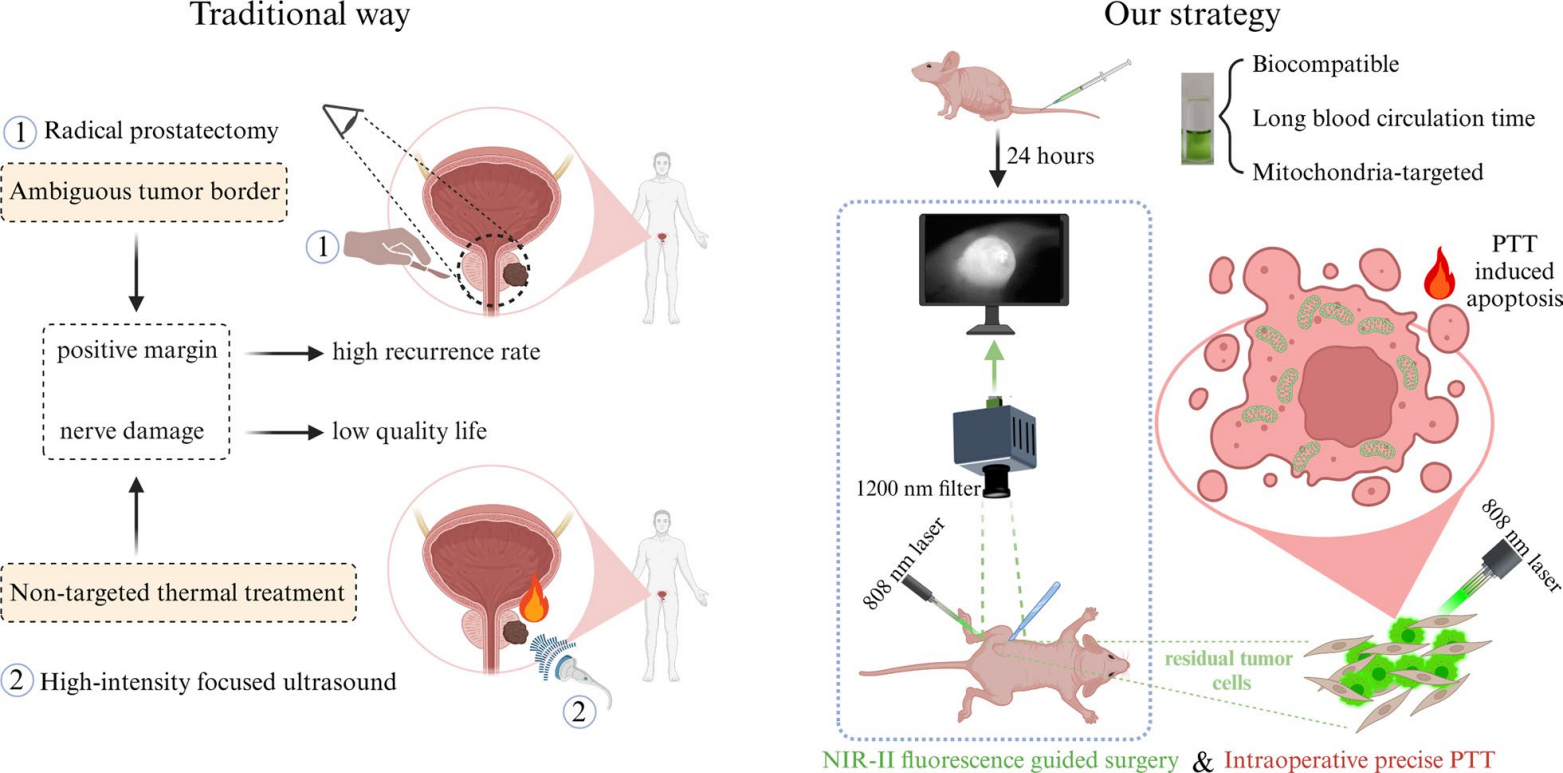

**Supplementary Information:**

The online version contains supplementary material available at 10.1186/s12951-024-02477-6.

## Introduction

Prostate cancer is one of the most commonly diagnosed malignancies in men worldwide [1]. Radical prostatectomy (RP) is the primary treatment option for most prostate cancer patients [2]. However, inadequate identification of the tumor boundary during RP can easily lead to positive surgical margins (PSMs), indicating a high risk of recurrence [3, 4]. Patients with PSMs require postoperative radiotherapy or androgen deprivation therapy (ADT) to eliminate residual tumors, which inevitably leads to adverse effects, complications, and increased financial costs [5]. In addition, clinical studies have shown that despite the administration of cutting-edge ADT, the tumor eventually relapses as castration-resistant prostate cancer [6]. Ablation is a guideline-recommended modality for localized prostate cancer treatment [7]. High-intensity focused ultrasound has been reported to effectively eliminate tumors by inducing rapid temperature changes [8]. However, the efficacy of high-intensity focused ultrasound is compromised with large or deep tumors [9]. Similar to surgical treatments, temperature transduction cannot selectively target tumor cells [8, 10]. Additionally, the pursuit of maximal lesion removal may inadvertently damage critical structures such as the urethral or sexual nerves, resulting in urinary incontinence or erectile dysfunction [11]. Therefore, it is essential to develop an image-guided adjuvant therapy that can accurately identify residual lesions and kill tumor cells intraoperatively.

Near-infrared-II fluorescence imaging, due to its noninvasiveness, high sensitivity, and high temporal resolution, has been widely used in biomedical fields and its imaging effects are highly dependent on the optical properties of the fluorescent probe [12, 13]. Previously reported NIR-II probes mainly include quantum dots, transition metal nanomaterials, two-dimensional materials, and small molecule dyes (SMDs) [14–17]. Among them, the cyanine dye ICG is the only NIR fluorescent diagnostic drug approved by the FDA for clinical use [18, 19]. Several heptamethine cyanine SMDs with tumor-targeting properties have been described previously [20–23]. An image-guided photothermal ablation agent based on new indocyanine green (IR820) and superparamagnetic oxide was constructed as a preoperative neoadjuvant treatment for hepatocellular carcinoma [24]. Another photothermal hydrogel platform based on IR820 has been reported to prevent postoperative tumor recurrence [25]. These studies suggest that heptamethine cyanine SMDs have great potential for image-guided adjuvant therapy [26–28]. However, most investigators have only tracked tumor growth data in the short term and have not focused on long-term survival differences. Secondly, these efforts lacked protection of normal tissues in the pursuit of tumor eradication. Moreover, the complicated design and synthesis processes of these nanomaterials may raise biosafety concerns and result in high costs [29–31]. As highlighted earlier, relying solely on surgery or photothermal therapy (PTT) presents challenges in effectively eliminating tumor cells while preserving normal tissue [32]. Consequently, employing PTT for the intraoperative eradication of margin-positive cells has emerged as a viable alternative [33, 34]. Furthermore, intraoperative photothermal intervention allows timely and minimally invasive tumor eradication [35, 36]. In particular, the integration of a SMD with NIR-II guided surgery and PTT for precise RP intraoperative adjuvant therapy has rarely been explored.

Herein, four heptamethine cyanine SMDs are described. Due to the high hydrophobicity of SMDs, the biocompatible theranostic nanocomposites were prepared by simply mixing the SMD and a solution of bovine serum albumin (BSA). The fluorescence performance and photothermal stability of SMD@BSA nanocomposites were investigated. In addition, the blood circulation time of these nanocomposites in vivo and preferential accumulation within tumors have been well explored. Therefore, a novel dual-function agent for fluorescence imaging and photothermal therapy in tumor cells, CY7-4, was selected and evaluated based on its optical and targeting properties. Ultimately, CY7-4 was used to identify primary and metastatic tumors. Such sensitive NIR-II fluorescence image-guided PTT accurately outlined the minimal residual prostate tumor foci and precisely ablated the residual tumor cells, thus completely inhibiting recurrence. Based on this novel agent, we propose a new method for the precise intraoperative treatment of prostate cancer, which has great potential for clinical translation.

## Experimental section

### Materials and methods

Four cyanine dye derivatives were obtained from QiLan Co. (Shenzhen, China). Indocyanine green (ICG, I2633, 50 mg) and bovine serum albumin (BSA, A1933, ≥ 98%) were purchased from Aldrich. DMEM, RPMI 1640, phosphate-buffered saline (PBS), and fetal bovine serum (FBS) were obtained from Gibco Co. (Shanghai, China). All reagents were used as received without further purification.

### Synthesis and characterization

The SMD@BSA nanocomposites were prepared according to previously reported methods. Typically, 20 μL of CY7-4 in DMSO (10 mM) was added to a BSA solution (the optimized dye-to-BSA mass ratio was 1:100) under vigorous vortexing and shaken overnight. The final solution was passed through a 0.22 μm syringe filter and stored at 4 ℃ for further use. Unless otherwise specified, all cellular and animal experiments were based on SMD@BSA nanocomposites rather than free SMD.

### Lentivirus, cell lines, and animals

The RM-1, HK2 and P69 cell lines were provided by the Institute of Urology, Union Hospital, Tongji Medical College, Huazhong University of Science and Technology. L-O2 cells and HUVECs were kind gifts from Dr. Jiao Xie. Luciferase lentivirus was obtained from Genechem (Shanghai, China). The luciferase-expressing cell line RM-1/Luc was constructed according to the manufacturer’s instructions. RM-1, RM-1/Luc, P69, and L-O2 cells were cultured in RPMI 1640 medium in a 37 ℃ incubator filled with 5% CO_2_. HUVECs and HK2 cells were cultivated in DMEM. All media were supplemented with 10% FBS and 1% streptomycin/penicillin (MIKX, China). Male C57 and BALB/c nude mice weighing 20 g were purchased from Zhuhai BaiShiTong Bio-Technology Co., Ltd. All animal experiments were conducted according to the guidelines of the National Institutes of Health, and the procedures were approved by the Animal Study Committee of the Shenzhen Institutes of Advanced Technology, Chinese Academy of Sciences. During surgery, the mice were anesthetized with 2.5% isoflurane in oxygen.

### In vitro cellular uptake

To evaluate dose-dependent cellular uptake, RM-1 cells were seeded in 6-well plates (1 × 10^5^ cells per well in 2 mL of RPMI 1640 medium). After 24 h, the medium was replaced with fresh medium containing 0–40 μM CY7-4, and the cells were incubated for 30 min. The free dye was removed by washing the cells 3 times with PBS before the addition of trypsin. The collected cells were imaged with an NIR-II camera in a 1.5 mL centrifuge tube and then resuspended in PBS. The cell suspensions were lysed by sonication on an ice bath (25 W, 3 s/3 s, 35%). Absorption spectra (in the range of 400–900 nm) were acquired for each final solution with a UV–vis spectrophotometer. To evaluate time-dependent cellular uptake, cells incubated with 20 μM CY7-4 for 0–120 min were collected for further analysis.

### Endocytosis and subcellular localization

For endocytosis analysis, RM-1 cells were pretreated with 50 μM amiloride or 7.5 mM MβCD for 1 h and then incubated with 10 μM CY7-4 for 20 min before observation via CLSM. For mitochondrial colocalization, cells were seeded in confocal dishes (0.5 × 10^5^ cells per dish in 2 mL of RPMI 1640). After incubation overnight, 100 nM Mito Tracker (Beyotime, China) was added to the medium. Thirty minutes later, the medium was replaced with fresh RPMI 1640 containing CY7-4 (10 μM), followed by staining with 50% (v/v) Hoechst 33342 (Beyotime, China) for another 30 min. Free dye was removed by gently washing the cells with PBS.

### Biodistribution

Eight-week-old C57 mice implanted with tumors were intravenously injected with CY7-4 or ICG (1 × 10^–6^ mol kg^−1^) (n = 6). An equal volume of PBS was injected intravenously into control mice. Fluorescence images were acquired from half of the mice at 0.5, 2, 6, 24, 48, and 72 h postinjection by an IVIS imaging system (λ_ex_ = 740 nm, λ_em_ = 840 nm, exposure time: auto). The remaining mice were sacrificed 24 h after intravenous administration, and the organs (heart, liver, spleen, lung, kidney, stomach, and bowel) were imaged in a flat dish. The fluorescence intensity of each image was quantified via Living Image software using ROIs to circle the region of interest.

### Cellular photothermal therapy

Calcein AM/PI and TUNEL staining were conducted according to the product manual. In brief, 1 μM calcein AM and 2 μM PI were added sequentially to the cells for 30 min of incubation. CLSM images for each group were acquired with the same settings (λ_ex_ = 488 nm, λ_em_ = 499–525 nm for calcein AM; λ_ex_ = 561 nm, λ_em_ = 571–625 nm for PI). Cells were treated with 4% paraformaldehyde for 30 min before TUNEL staining. The TUNEL assay solution consisted of the TDT enzyme and FITC-12-dUTP labeling mix. DNase I and TUNEL assay solution without TDT served as positive and negative controls, respectively.

### NIR-II fluorescence-guided surgery

Before the operation, CY7-4 (1 μmol kg^−1^) was injected intravenously. 24 h later, the tumor and suspected metastases were surgically excised under NIR-II fluorescence guidance (λ_ex_ = 808 nm, exposure time: 100 ms, filter: 1200 LP). HE staining was utilized to determine the malignancy of each lesion.

### NIR-II fluorescence-guided photothermal therapy

After the tumor model was established, the mice were randomly divided into five groups: the PBS nonirradiated group, the PBS irradiation group, the ICG irradiation group, the CY7-4 nonirradiated group, and the CY7-4 irradiation group (n = 5). In each laser treatment group, the mice received continuous laser irradiation (808 nm, 2.5 W cm^−2^) for 10 min after intravenous injection of the agent (ICG/CY7-4, 1 μmol kg^−1^). Laser irradiation alone and injection of CY7-4 or PBS served as controls. Changes in the temperature of the lesion under laser irradiation were recorded in real-time with a handheld thermal imaging camera (FLUKE). Two weeks later, the mice were sacrificed, and the tumors were removed and weighed.

### NIR-II fluorescence-guided photothermal therapy during surgery

BALB/c nude mice bearing RM-1/Luc tumors were divided into different groups (n = 5). In the combined surgery and photothermal treatment group, a small portion of the tumor lesion was artificially left in situ in the mice to mimic a clinically positive margin. The difference between radical and partial resection is whether suspicious lesions detected by fluorescence imaging are removed.

### Evaluation of the therapeutic effect and biocompatibility

Changes in the tumor size and body weight of each mouse after treatment were documented regularly. For survival analysis, tumor growth up to 500 mm^3^ or weight loss of more than 20% was defined as the endpoint of observation. Bioluminescence was employed to assess residual tumors and tumor recurrence.

To investigate toxicity in vivo, the mice were randomly divided into three groups and injected with an equal volume of CY7-4, ICG (2.5 μmol kg^−1^), or NaCl (0.9% w/v) (n = 5). Blood samples were obtained from the mice by removing the eyeballs 24 h after intravenous administration, and the serum was separated by centrifugation at 5000 × g for 10 min. Terminal blood collection was necessary to obtain enough serum for biochemical analysis and routine blood testing. After that, the mice were euthanized, and the whole operation was carried out under anesthesia. Liver and kidney function indicators were determined using an automatic biochemical analyzer (Chemray 800, Rayto). The body weights and physical behaviors of the mice in the other groups were monitored for two weeks after injection of different agents. After that, the mice were sacrificed, and the heart, liver, spleen, lung, kidney, and intestines were harvested for histopathological analysis.

### Statistical analysis

All the data were analyzed and plotted with GraphPad Prism 8 software. The data are presented as the mean ± sd. Differences between two groups were analyzed by Student’s t-test. The Mantel‒Cox test was utilized to analyze differences in survival between two groups. The data were compared to those of the PBS group unless specifically noted. A value of *P* < 0.05 was considered to indicate statistical significance.

## Results and discussion

### Characterization of the NIR fluorophores

**Fig. 1:**
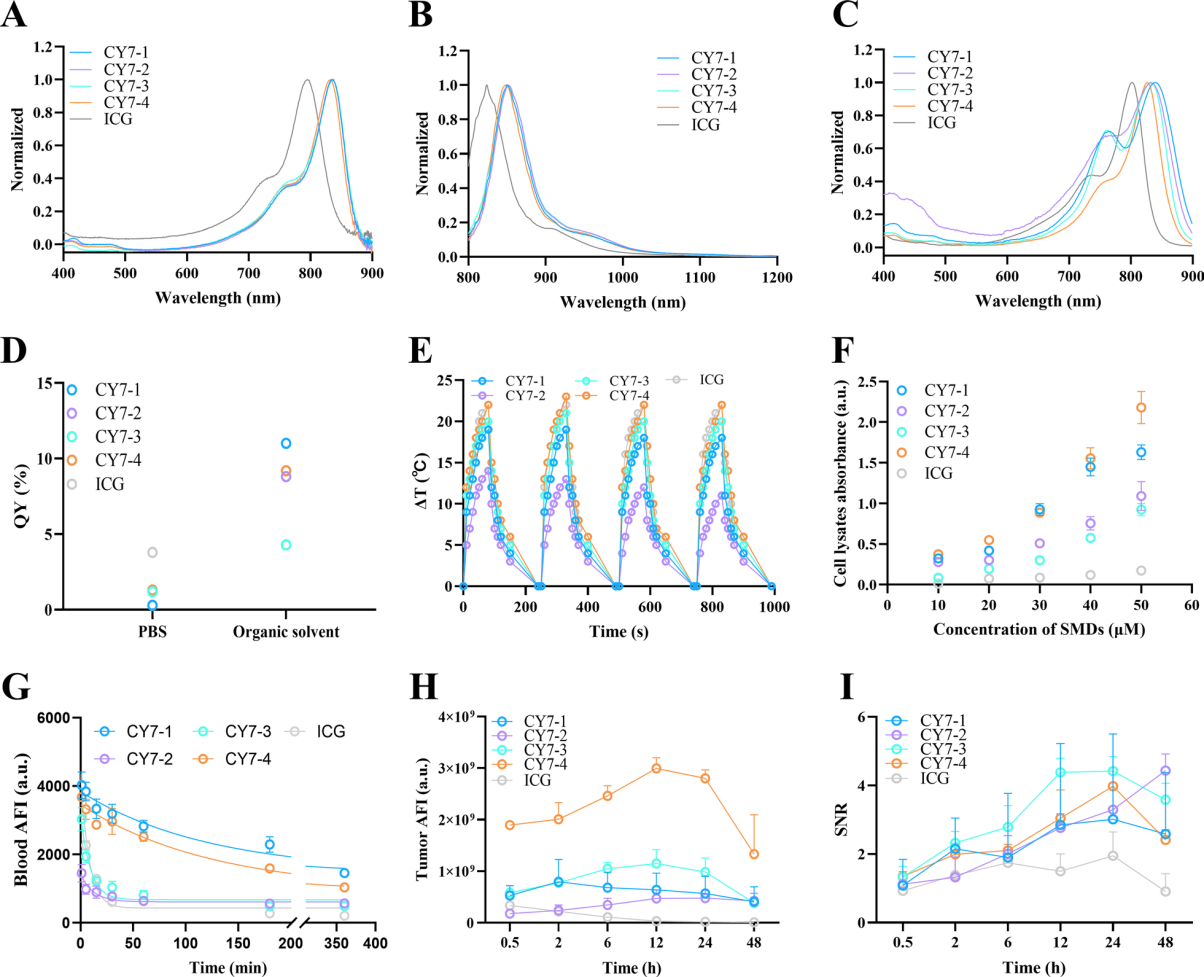
Characterization of the SMDs. **A**, **B** Normalized absorption and fluorescence spectra of the SMDs in DMSO. **C** Normalized absorption spectra of the SMD@BSA in PBS. **D** Absolute quantum yields of the SMDs in PBS or organic solvents (CY7-1, CY7-2, and CY7-4 in dichloromethane; CY7-3 and ICG in ethanol). **E** Heating and cooling curves of the SMDs after four cycles (100 μM, 808 nm laser irradiation, 1 W cm^−2^). **F** Peak absorbance values of the cell lysates after incubation with different concentrations of SMDs. **G**–**H** Quantification of average blood and tumor fluorescence intensities at different time points after the mice were i.v. injected with the SMDs (1 μmol kg^−1^). The blood samples were imaged with an NIR-II camera (λ_ex_ = 808 nm, 100 ms, 1200 nm long-pass filter), and the tumors were imaged via an IVIS (n = 3). **I** Mouse tumor signal-to-noise ratios in (**H **)

The optical spectra of these dyes in DMSO are shown in Fig. 1A, B. The maximum absorbance and emission wavelengths were approximately 838 nm and 852 nm, respectively. Due to the hydrophobicity of the derivatives, biocompatible BSA-bound nanocomposites were prepared for in vivo experiments, as shown in Additional file 1: Figure S1A [37]. A mass ratio of 100:1 was considered appropriate for BSA binding to SMD (Additional file 1: Figure S1B–D). The normalized absorption spectra and photographs of each nanocomposite in PBS after optimization are shown in Fig. 1C and Additional file 1: Figure S1A, respectively. It has been reported that the affinity of a SMD for albumin is correlated with the chemical structure of the SMD, including the small size of each side group and relatively high hydrophobicity [38]. The hydrated particle size of the prepared CY7-4 nanocomposites and pure BSA were around 7.97 nm and 6.82 nm, respectively (Additional file 1: Figure S2). The absorption spectrum of the CY7-4 nanocomposites did not show any characteristics consistent with aggregation, which is critical for its photoluminescence performance both in vivo and in vitro. As shown in Fig. 1B, the longest emission was observed over 1000 nm, and the fluorescence intensity of CY7-4 was significantly stronger than that of ICG in the NIR-II window. Moreover, the higher quantum yield of CY7-4 did not compromise its photothermal properties. During NIR laser irradiation, the temperatures of these SMDs in PBS were repeatedly recorded four times, and a rapid temperature increase from 26 ℃ to 50 ℃ in 80 s was observed (Fig. 1E). More importantly, the in vivo imaging system (IVIS) results showed that the tumor fluorescence intensity and signal-to-noise ratio (SNR) of the mice injected with CY7-4 and CY7-3 were significantly greater than those of the other groups at all time points (Fig. 1H, I). In particular, the average tumor fluorescence intensity in the CY7-4 group was 147 times greater than that in the ICG group, which was attributed to the high affinity of CY7-4 for tumor cells (~ 5.6-fold) and its long half-life (87.1 min vs. 5.5 min) (Fig. 1F, G).

### Cell uptake and subcellular colocalization

**Fig. 2:**
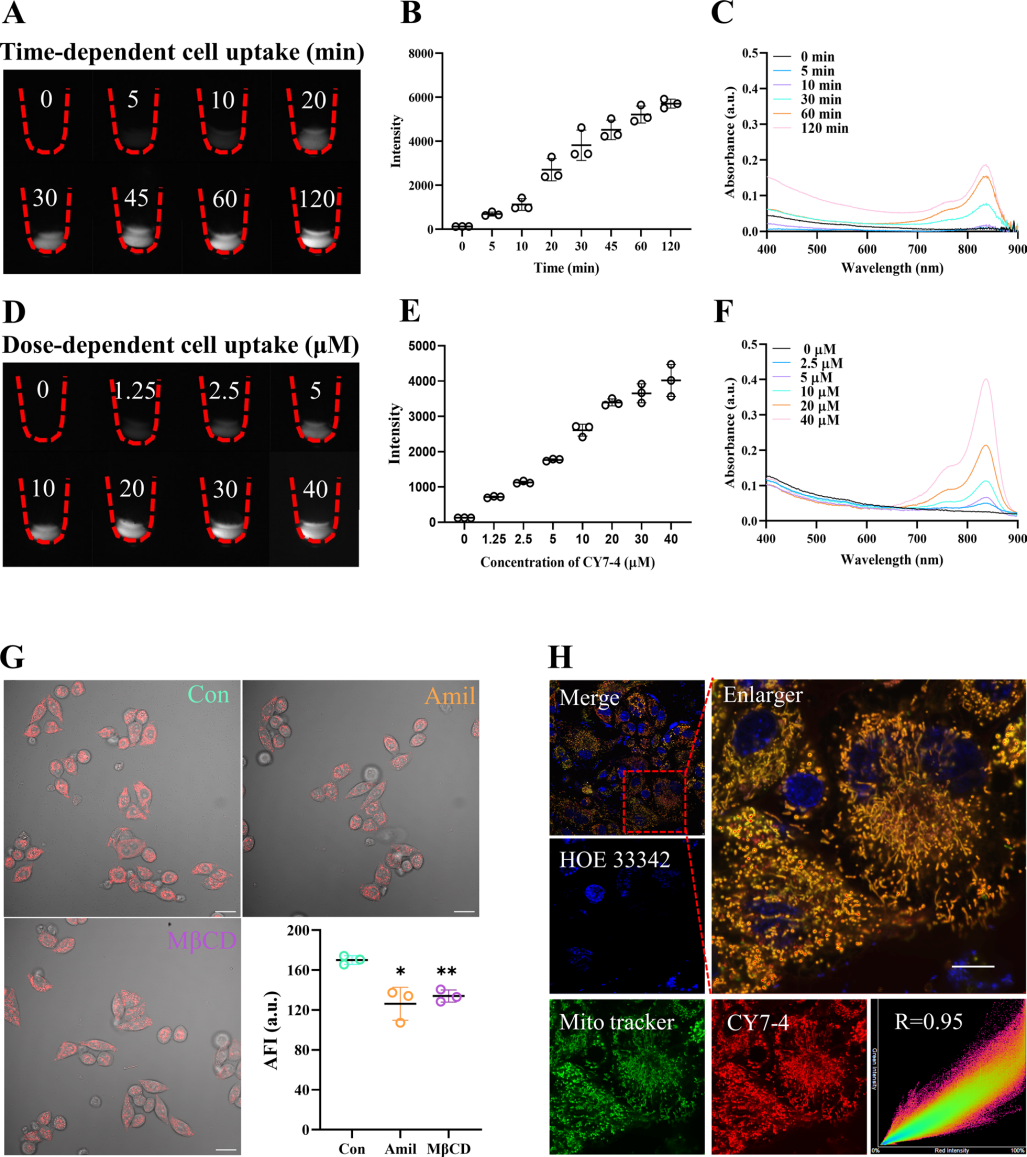
Cell uptake and subcellular colocalization. **A**–**B** Images of collected RM-1 cells incubated with 20 μM CY7-4 for different durations (λ_ex_ = 808 nm, 100 ms, 1200 nm long-pass filter) and the quantified fluorescence intensity. **C** Absorption spectra of the cell lysates in (**A**). **D**–**E** Images of RM-1 cells incubated with different concentrations of CY7-4 for 30 min (λ_ex_ = 808 nm, 100 ms, 1200 nm long-pass filter) and the quantified fluorescence intensity. **F** Absorption spectra of the cell lysates in (**D**). **G** CLSM images of living RM-1 cells stained with 10 μM CY7-4 after 60 min of pretreatment with 50 μM Amil or 7.5 mM MβCD and quantification of the average fluorescence intensity. Scale bars: 20 μm. *: *P* < 0.05, **: *P* < 0.01 **H** CLSM images of living RM-1 cells stained with 10 μM CY7-4 (red) after 30 min of pretreatment with Mito Tracker (green) and incubation with Hoechst 33,342 (blue) for 30 min before imaging. Pearson coefficient: R = 0.95. Scale bar: 10 μm

To assess tumor targeting ability, NIR-I imaging was performed on prostate tumor-bearing C57 mice injected intravenously with the SMDs at different time points using the IVIS system. A signal-to-noise ratio (SNR) between the tumor and para-tumor of more than 2.5 was considered targeted accumulation [39]. Our in vivo imaging results revealed that these derivatives significantly preferentially accumulated in the tumor (Additional file 1: Figure S5). To investigate the tumor-targeting mechanisms, cellular uptake was quantified. The absorption spectroscopy results indicated that the absorbance of CY7-4 was nearly 10 times greater than that of ICG after incubation of the dye with tumor cells for the same period of time (Fig. 1F). The high affinity in vitro may be due to the positive charge and lipophilicity of CY7-4 [40]. Furthermore, Fig. 2A–F shows that CY7-4 was taken up in a time and dose-dependent manner. This explains why CY7-4 is favored for uptake by tumor cells after longer blood circulation times, leading to a stronger fluorescence signal. In addition to a long circulation time, tumor accumulation, tumor cell endocytosis, and drug release are essential for tumor-targeted drug delivery [41]. Figure 2G shows that pretreatment of RM-1 cells with amiloride (Amil, a micropinocytosis inhibitor) or methyl-β-cyclodextrin (MβCD, a caveolae-mediated endocytosis inhibitor) inhibited CY7-4 uptake. The subcellular localization of CY7-4 in RM-1 cells was then investigated (Fig. 2H). The fluorescence confocal microscopy images showed that the red fluorescence of CY7-4 perfectly overlapped with the green fluorescence of the commercial mitochondrial tracker (Mito-Tracker). This is consistent with previous reports that some positively charged cyanine dyes have the property of targeting negatively charged mitochondrial membranes [42–44]. These results indicated that CY7-4 was taken up via endocytosis and localized in mitochondria. Tumor cells have been reported to have greater endocytosis efficiency than normal endothelial cells, while excellent mitochondrial subcellular localization also reduces drug exocytosis and enhances the SNR [45].

### Pharmacokinetics and biosafety assessments

**Fig. 3:**
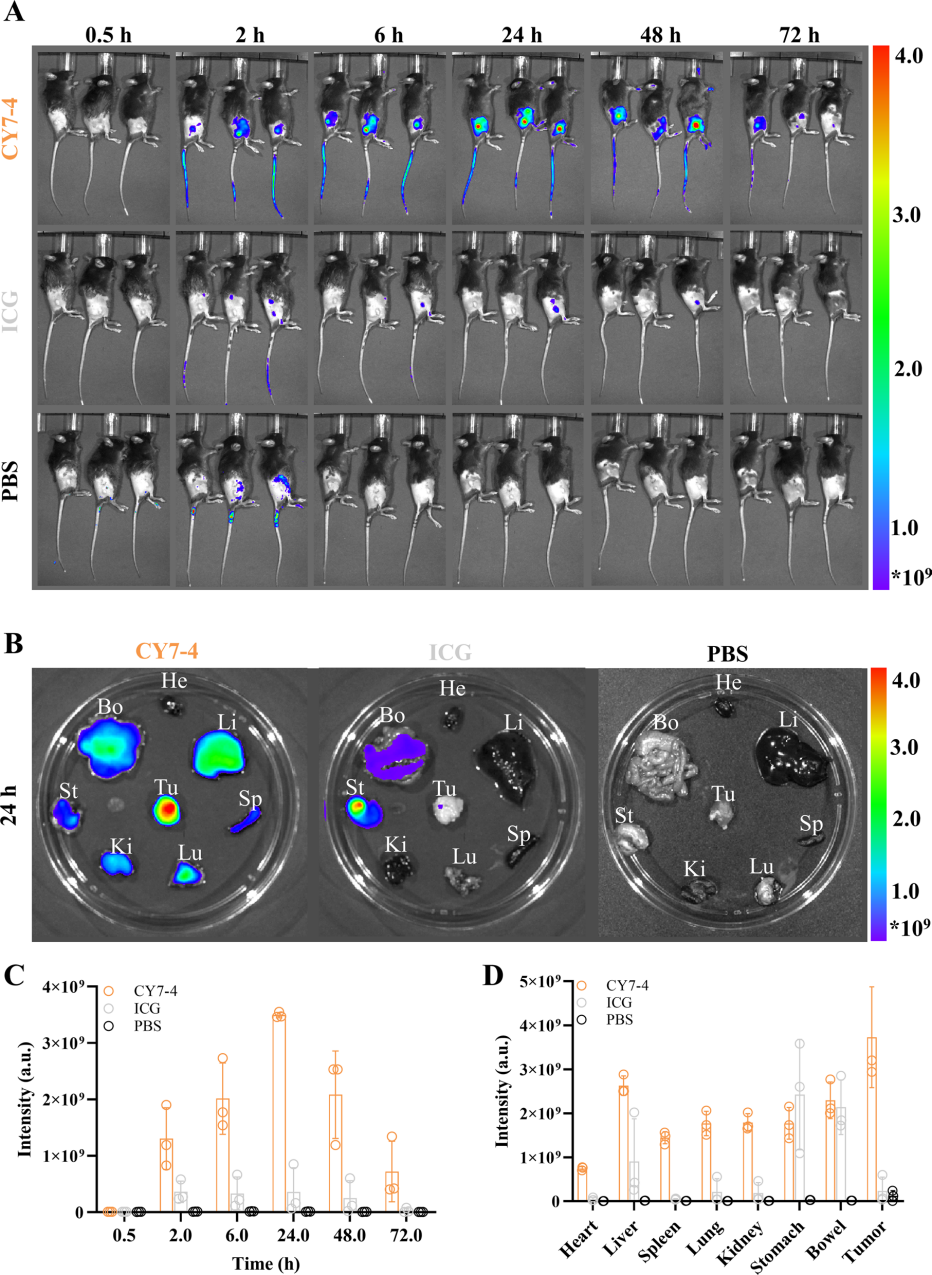
Tumor targeting and biodistribution of CY7-4 and ICG. **A** NIR-I images of mouse body tissues at different time points after i.v. injection of 1 μmol kg^−1^ CY7-4 or ICG. PBS served as a control; n = 3. **B** NIR-I images of mouse organs 24 h after i.v. injection of 1 μmol kg^−1^ CY7-4 or ICG (He: heart, Li: liver, Sp: spleen, Lu: lung, Ki: kidney, St: stomach, Bo: bowel, Tu: tumor). **C** Quantification of tumor fluorescence in the mice in (**A**) at the corresponding time points. **D** Quantification of the fluorescence intensity in the mouse organs in (**B**)

**Fig. 4:**
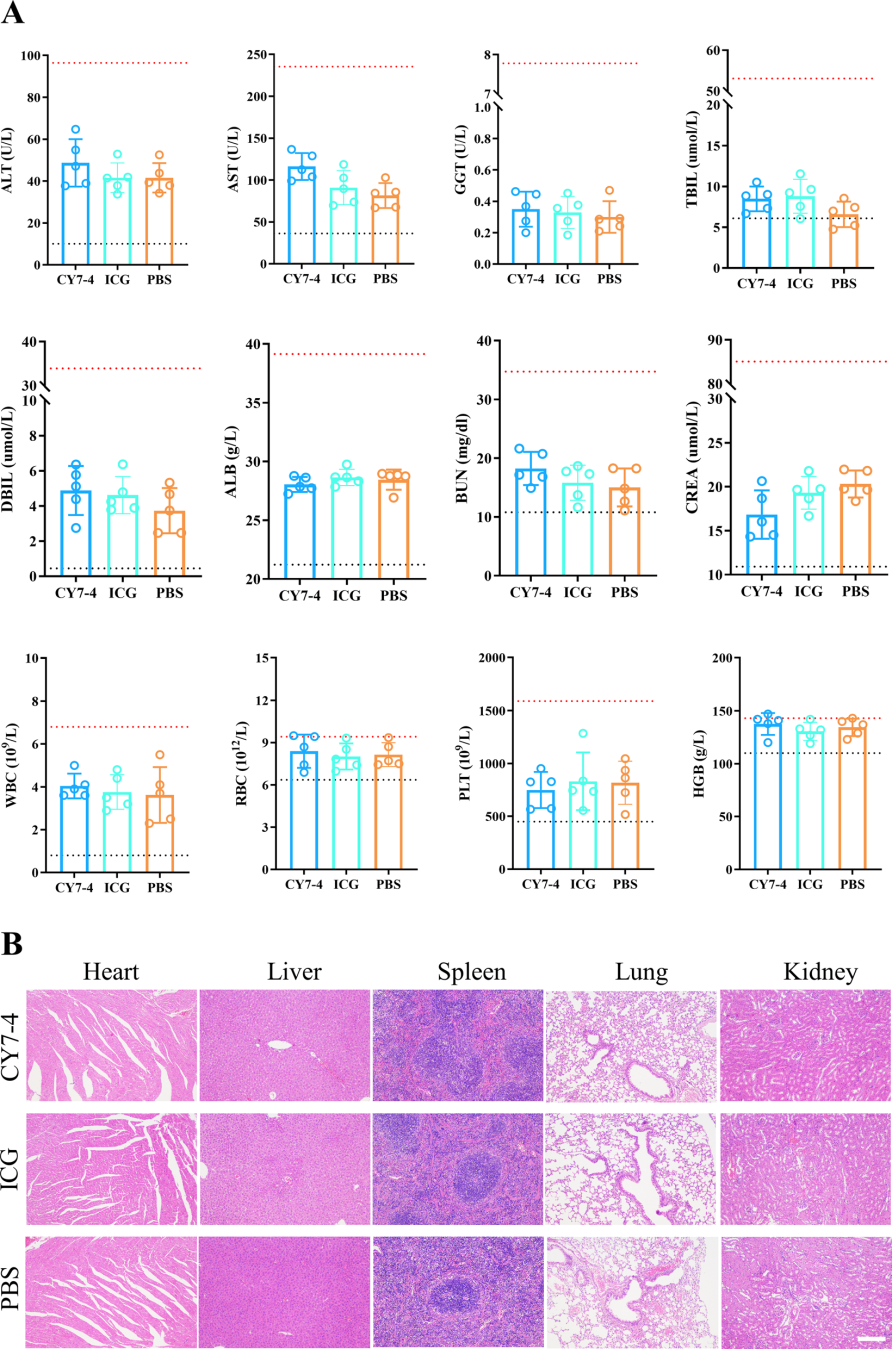
Biosafety assessment. **A** Blood biochemistry analysis and routine blood test results 24 h after i.v. injection of 2.5 μmol kg^−1^ CY7-4 and ICG. PBS served as a control; n = 5. The dotted lines represent the upper and lower values of the normal range for each metric. **B** Images of HE-stained slices of major organs from mice injected with CY7-4, ICG, or PBS prepared 2 weeks after treatment. Scale bar: 200 μm

The blood concentration profiles of all the SMDs in the mice are shown in Fig. 1G. The tumor mitochondria-targeting agent CY7-4 has a half-life of more than 2 h, while ICG is rapidly cleared. Therefore, we believe that the preferential accumulation of CY7-4 in tumors observed via NIR-I imaging and the extraordinarily enhanced fluorescence signal of CY7-4 in tumors may be related to its longer half-life and greater cellular uptake. The metabolic behavior of SMD@BSA nanocomposites were related to the degree of covalent bonding [38]. Based on these findings, CY7-4 was identified as a potential multifunctional small molecule for simultaneous cancer-targeted NIR-II imaging and PTT. The biodistribution and biosafety of CY7-4 and ICG were further investigated. Fluorescence images of the whole body at different time points and of organs at 24 h after agent injection are shown in Fig. 3A, B. Both agents were excreted by the liver and eliminated via the digestive system (Fig. 3D). The fluorescence quantification profiles showed a similar trend of tumor accumulation: the average fluorescence intensity of the tumors increased with increasing time after i.v. injection of the agents, reaching a maximum at 24 h (Fig. 3C). In the biosafety assessment, no significant cytotoxicity or hemolysis was observed even in the group receiving the highest dose (Additional file 1: Figures S6, 7). Likewise, the hepatic and renal functions and routine blood test results of these mice were within the normal range. HE staining did not reveal any congestion, edema, necrosis, or inflammation in the organs (Fig. 4A, B).

### Cell viability and in vitro photothermal therapy

**Fig. 5:**
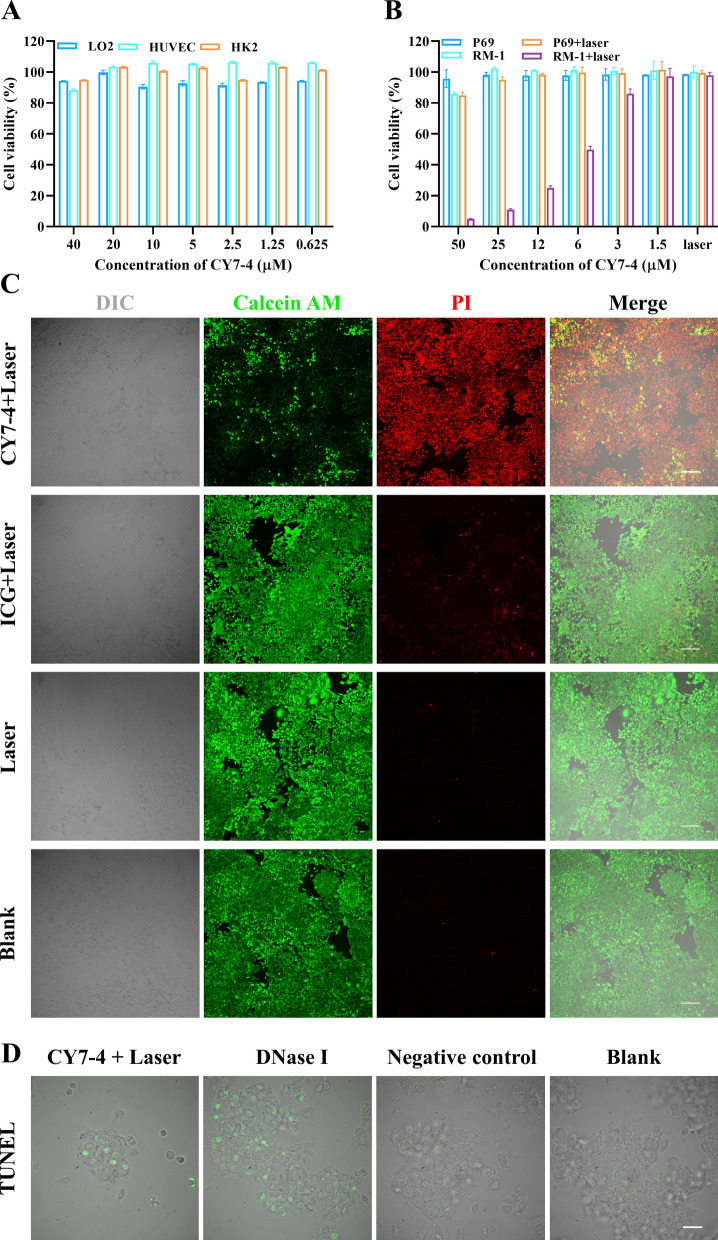
In vitro PTT with CY7-4. **A** Viability of L-O2 cells, HUVECs, and HK2 cells treated with different concentrations of CY7-4 for 12 h. **B** Viability of P69 and RM-1 cells treated with various concentrations of CY7-4 for 12 h with or without 808 nm laser irradiation (1 W cm^−2^, 5 min). **C** Fluorescence images of RM-1 cells treated with 20 μM CY7-4 or ICG with 808 nm laser irradiation and calcein-AM/PI costaining; laser irradiation alone served as a negative control (1 W cm^−2^, 5 min). Red channel: dead cells; green channel: living cells; scale bar: 200 μm. **D** Fluorescence images of RM-1 cells treated with 20 μM CY7-4 for 30 min (808 nm laser irradiation, 1 W cm^−2^, 5 min) stained with TUNEL. The DNase I-treated group served as a positive control. Scale bar: 20 μm

CCK-8 assays with normal human cell lines (L-O2 cells, HUVECs, and HK2 cells) were performed to evaluate the effect of CY7-4 on cell viability in vitro. Figure 5A shows that CY7-4 did not significantly inhibit cellular activity at fourfold the experimental concentration. We next determined the viability of RM-1 and P69 cells by CCK-8 assays after 2 h of incubation with CY7-4 followed by laser irradiation (808 nm, 1 W cm^−2^). The results showed that cell viability decreased in a CY7-4 dose-dependent manner (from 100% to nearly 0% at 50 μM). In contrast, the groups that did not receive irradiation exhibited no significant decrease in viability. Similarly, the viability of normal prostate cells (P69 cells) after incubation and irradiation at the same dose was not significantly impaired (Fig. 5B). We next investigated the mechanism of PTT via CLSM. After 2 h of incubation with 20 μM CY7-4 and 5 min of NIR irradiation (808 nm, 1 W cm^−2^), an apoptotic ratio > 95% was observed in the CY7-4 + laser group. Although ICG showed photothermal effects similar to those of CY7-4 in tubes, ICG was unable to achieve effective PTT due to poor cellular uptake (Fig. 5C). The TUNEL results (Fig. 5D) also show that there were more apoptotic cells in the CY-4 + laser group. Taken together, these data indicated that CY7-4 mediated cancer cell apoptosis following NIR irradiation in a dose-dependent manner.

### NIR-II imaging and fluorescence-guided surgery

**Fig. 6:**
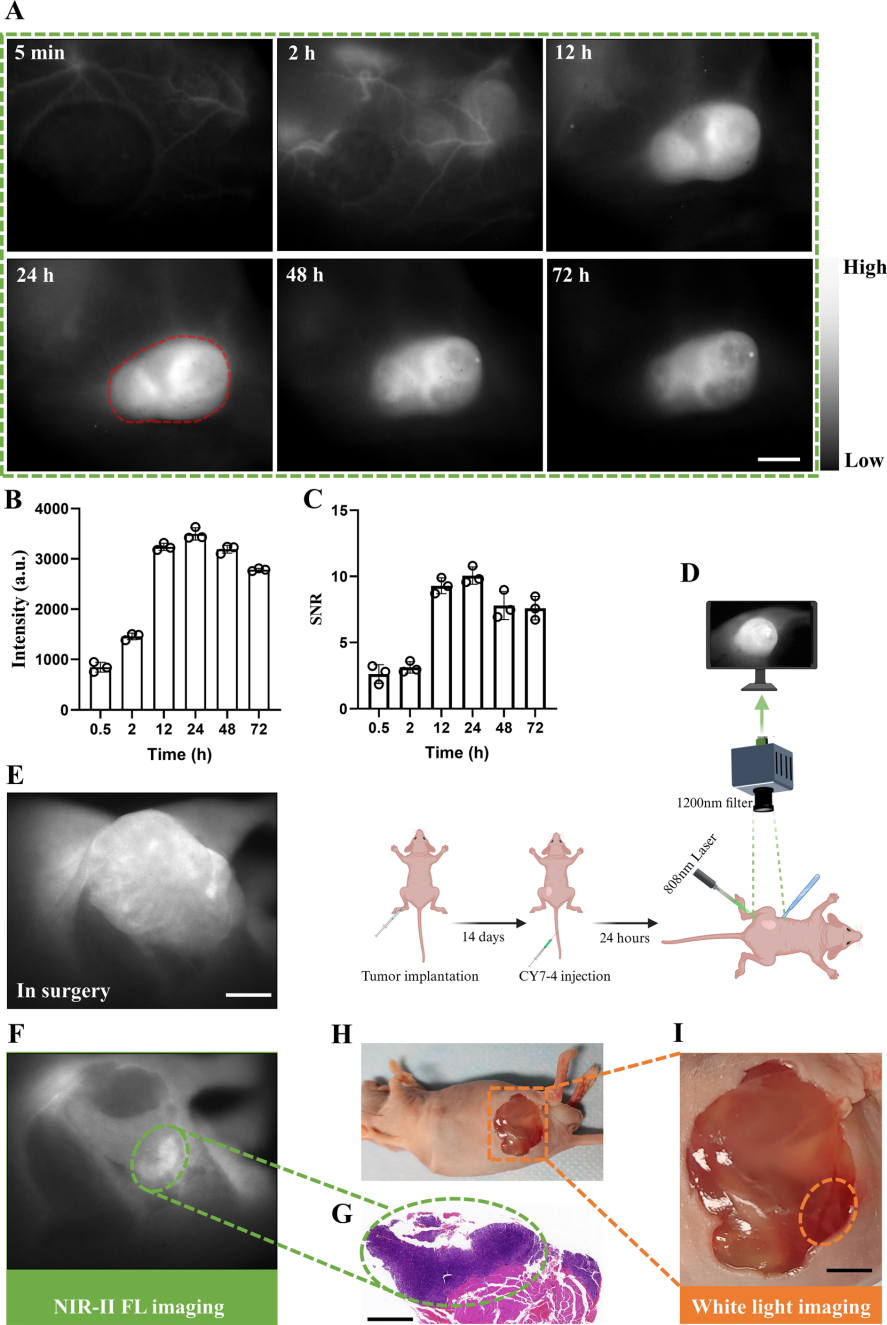
Tumor diagnosis and NIR-II fluorescence-guided surgery. **A** An NIR-II camera was used to acquire representative images of BALB/c nude mice at different time points (λ_ex_ = 808 nm, 100 ms, 1200 nm long-pass filter) after i.v. injection of CY7-4 (1 μmol kg^−1^), n = 3. Red circle: tumor borders; scale bar: 2 mm. **B**, **C** Tumor fluorescence intensities and quantification of the SNRs at the corresponding time points. **D** Schematic of NIR-II fluorescence-guided surgery. **E** Image of the RP process under NIR-II guidance; scale bar: 2 mm. **F**, **G** Suspicious lesions were detected with NIR-II fluorescence imaging and confirmed pathologically by staining sections with HE. Scale bar: 500 μm. **H**, **I** Photograph of a mouse’s body and an enlarged image of the surgical area around the tumor. The suspicious lesions in **F** were not visible under white light. Scale bar: 2 mm

NIR-I imaging with the IVIS system reveals drug metabolism kinetics and tumor-targeted accumulation. However, this technique failed to facilitate fluorescence-guided surgical treatment due to its low resolution. Hence, we explored the tumor-targeting properties of CY7-4 in BALB/c nude mice and fluorescence-guided RP using our NIR-II imaging system. The imaging device is shown in Additional file 1: Figure S4B. The imaging range was delineated as FOV = (22 mm × 17.6 mm) to achieve higher spatial and temporal resolution. As expected, tumor vessels were visualized immediately, and this signal enhancement lasted for more than 2 h due to the long half-life of CY7-4 (Additional file 1: Figure S8). Such distinguishable vascular distribution may help to differentiate between benign and malignant lesions and minimize intraoperative bleeding [46, 47]. Consistent with the NIR-I imaging results, the tumor signal intensity increased after compound administration and peaked in the 24th h (Fig. 6A). Moreover, Fig. 6B shows that the high signal intensity was maintained for more than 2 days, providing a long window for intraoperative fluorescence-guided surgery. More excitingly, the contrast between the tumor and paraneoplastic tissue improved from 4 in the NIR-I to nearly 10, providing precise guidance for sectioning the lesion and photothermal ablation of the cancer cells (Fig. 6C). No significant signal was detected in the tumor region of ICG-injected (control) mice (Additional file 1: Figure S8A). NIR-II-guided RP was performed on RM-1 tumor-bearing BALB/c nude mice (Fig. 6E). As depicted in Fig. 6F, G, a suspicious fluorescent lesion was excised and pathologically confirmed to be residual tumor tissue, which was not identified by the naked eye intraoperatively (Fig. 6H, I). In addition, lymph node metastatic lesions were effectively recognized (Additional file 1: Figure S9).

### Fluorescence-guided photothermal therapy

**Fig. 7:**
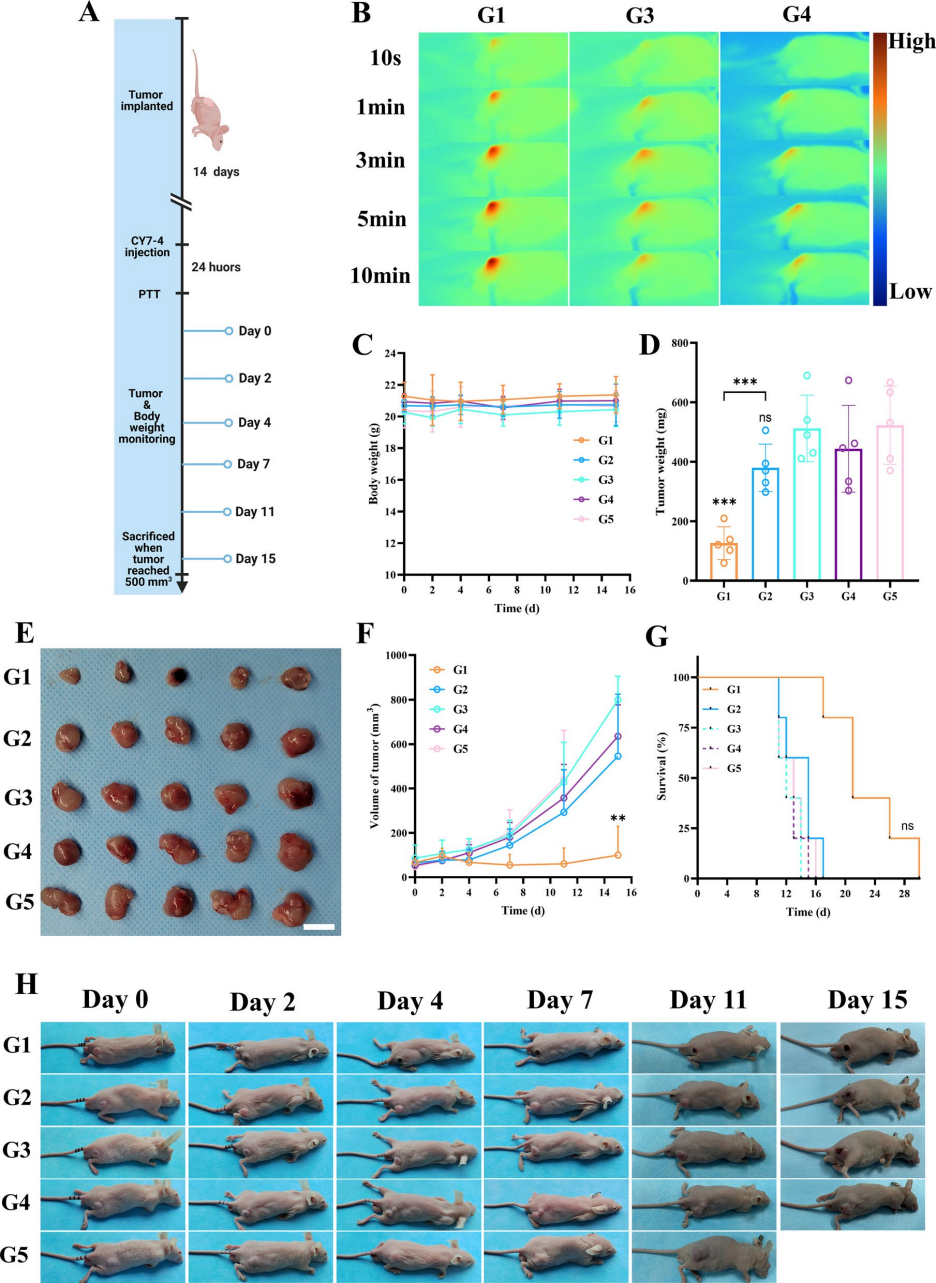
Fluorescence-guided photothermal therapy. **A** Schematic of fluorescence-guided PTT. G1: injected with 1 μmol kg^−1^ CY7–4 + irradiation (808 nm, 2.5 W cm^−2^); G2: injected with 1 μmol kg^−1^ CY7-4; G3: injected with 1 μmol kg^−1^ ICG + irradiation; G4: injected with PBS + irradiation; and G5: injected with PBS. **B** Infrared thermographic images of mice in G1, G3, and G4. G1: n = 10; other groups: n = 5. **C** Body weights of mice that received different treatments, n = 5. **D** Tumor weights on Day 15 after receiving different treatments, n = 5. **E** Representative images of tumors from mice in each group 15 days after treatment, n = 5. Scale bar: 10 mm. **F** Changes in tumor volume in the different groups as a function of time, n = 5. **G** Survival rate in each group; n = 5. The experiment started with the establishment of the tumor model. **H** Representative mouse photos in each group showing the changes in tumor size from Day 0 to Day 15 after the start of treatment. The data are presented as the mean ± sd. The data were compared to those of the PBS group unless specifically noted. ns: no significant difference, *: *P* < 0.05, **: *P* < 0.01, ***: *P* < 0.001, ****: *P* < 0.0001

To avoid interference from melanin in the skins of C57 mice, in vivo photothermal therapy was performed in subcutaneous tumor-bearing BALB/c nude mice [48] (Additional file 1: Figure S4A). As shown in Fig. 7B, the temperature of the mice in the CY7-4-injected group reached 45 ℃ after 2 min of irradiation and 55 ℃ after 5 min of continuous irradiation, which was sufficient to ablate the tumor [49]. In contrast, the temperature in the PBS- and ICG-injected irradiation groups increased to 40 and 43 ℃, respectively, after 10 min of irradiation. After that, the whole-body status and body weight of each mouse were recorded periodically, and representative photographs are displayed in Fig. 7H. The results showed a rapid increase in tumor size in the PBS- and ICG-injected groups, in which no mice survived after two weeks of treatment (Fig. 7F). In contrast, the tumor volume of the mice in the CY7-4 irradiation group rapidly decreased after laser irradiation. Both the tumor volume and tumor weight in the CY7-4 irradiation group were significantly different from those in the other groups on Day 15 (Fig. 7D). However, another group of mice that received CY7-4 injections as well as irradiation showed rapid tumor growth after 2 weeks of treatment, and ultimately, no mice survived after 1 month (Fig. 7G). Although the short-term data showed that the CY7-4 irradiation group had a significantly better treatment response, the survival analysis showed no significant difference among the five groups. We believe that this may be due to recurrence of residual tumor cells, suggesting that it is difficult to eliminate the entire lesion with a single photothermal treatment.

### Fluorescence-guided surgery combined with photothermal therapy

**Fig. 8:**
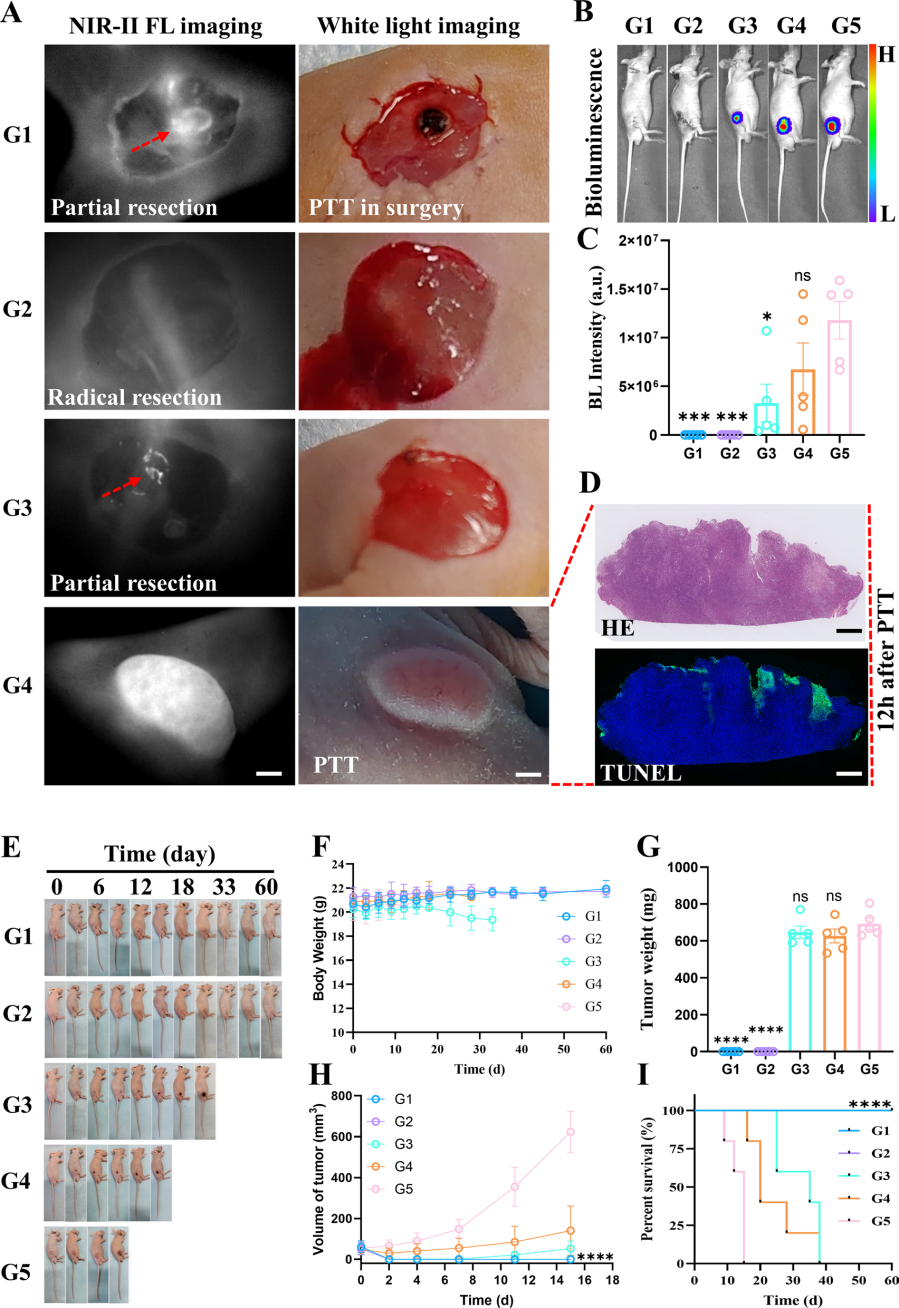
NIR-II fluorescence-guided surgery combined with intraoperative photothermal therapy.** A** Representative NIR-II fluorescence images and white light images from each group. G1: partial resection combined with PTT; G2: radical resection; G3: partial resection; G4: PTT alone; and G5: PBS. Red arrow: residual tumor; scale bar: 1 mm. **B**, **C** Bioluminescence images and quantification of signal intensity each group 24 h after the start of treatment. **D** HE- and TUNEL-stained sections prepared at 24 h after irradiation in G4. Scale bar: 500 μm. **E** Representative photos of mice in each group showing the changes in tumor size from Day 0 to Day 60 after the start of treatment. **F** The body weights of the mice that received different treatments, n = 5. **G** Tumor weights on Day 60 after receiving different treatments, n = 5. **H** Changes in tumor volume in the different groups as a function of time, n = 5. **I** Survival rate in each group, n = 5. The experiment started with the establishment of the tumor model. The data are presented as the mean ± sd. The data were compared to those of the PBS group unless specifically noted. ns: no significant difference, *: *P* < 0.05, **: *P* < 0.01, ***: *P* < 0.001, ****: *P* < 0.0001

As shown in Fig. 8A, the mice were randomly divided into five groups: partial resection combined with PTT treatment (G1), radical resection (G2), partial resection (G3), PTT alone (G4), and PBS alone (G5) (n = 5). Bioluminescence imaging was performed 24 h after surgery to confirm the presence of residual tumor cells (Fig. 8B). Figure 8C shows that the bioluminescence in Groups G1 and G2 was barely detectable, indicating that the tumor was removed. The luminescence intensity in the G3 group was also significantly different from that in the G5 group, suggesting that partial excision could effectively reduce the tumor burden. However, the tumor killing effect of PTT alone was not significantly different from that of PBS (Group G5). Figure 8D suggests that the efficacy of PTT alone may be limited by the light penetration depth, which can only mediate superficial tumor cell apoptosis. After treatment, each mouse's whole-body status and body weight were recorded periodically, and representative photographs are displayed in Fig. 8E and Additional file 1: Figure S10. The results showed a rapid increase in tumor volume in the PBS group. In contrast, tumor growth was significantly inhibited in the other groups after two weeks of treatment (Fig. 8H). However, the tumors in the partial resection group and in the PTT alone group recurred within 1 month. Survival was significantly prolonged in the combination treatment and radical surgery groups (Fig. 8I). Interestingly, bleeding and incision suture length were significantly shorter in the combined treatment group than in the radical surgery group (Additional file 1: Figure S11). In conclusion, the same therapeutic efficacy was achieved with fluorescence-guided partial resection combined with intraoperative PTT and radical surgery. The optical and targeting properties of CY7-4 were fully utilized during combination therapy, and more minimally invasive precision treatments are expected to be developed.

## Conclusions

In this study, we proposed a new precise treatment strategy for prostate cancer, that aims to reduce the tumor recurrence rate by guiding surgical resection with near-infrared fluorescent probes targeting the tumor combined with intraoperative photothermal therapy. Surgical resection of most tumor tissues overcomes the disadvantage of insufficient light penetration depth, while photothermal therapy induced by light can further selectively kill probe-enriched cells. This strategy is particularly suitable for prostate cancer, brain cancer, and other tumors that are adjacent to important nerves and blood vessels. We expect that this concept should be validated based on more targeted probes in more types of cancer to accelerate its clinical translation.

### Supplementary Information

**Additional file 1: Figure S1.** (A) Schematic illustration of CY7-4 nanocomposite preparation process. (B) The absorption spectra of CY7-4 nanocomposite in PBS with different mass ratio. (C-D) White light and NIR-II images of CY7-4 nanocomposite with different mass ratio and NIR-II fluorescence quantification. **Figure S2.** Hydrated particle size of CY7-4 nanocomposites (A) and pure BSA (B). (C) Zeta potential of free CY7-4. **Figure S3.** Physicochemical stability of free CY7-4 in DMSO and CY7-4 nanocomposites. Changes in the appearance of prepared free CY7-4 solutions (A) and CY7-4 nanocomposites (B) over a week at room temperature. Absorption spectra of prepared free CY7-4 solutions (C) and CY7-4 nanocomposites (D) at different time points. No precipitation or decrease in peak absorbance value was observed. **Figure S4.** (A) Photograph of NIR-II fluorescence guided PTT process. (B) Blood samples imaging with a homebuilt NIR-II imaging system. **Figure S5.** The IVIS imaging system acquired representative images of C57 mice at different time points (λ_ex_ = 740 nm, λ_ex_ = 840, auto) after i.v. injected with different SMD@BSA nanocomposite (A: CY7-1, B: CY7-2, C: CY7-3, D: CY7-4, E: ICG, 1 μmol kg^-1^), n = 3. **Figure S6.** (A-B) Representative photo of red blood cells incubated with different concentrations of CY7-4 and absorbance values at 570 nm. **Figure S7.** (A-C) Cell viability of L-O2, HK2, and HUVEC cells treated with different concentrations of CY7-4 for 12 or 24 hours. **Figure S8.** (A) The NIR-II camera acquired representative images of C57 mice at different time points (λ_ex_ = 808 nm, 100 ms, 1200 nm long-pass filter) after i.v. injected with CY7-4 or ICG (1 μmol kg^-1^), n = 3. (B-C) Tumor fluorescence intensity and SNR quantification of CY7-4 injected mice at corresponding time points. **Figure S9.** (A-B) NIR-II imaging of suspicious metastases in prostate tumor bearing BALB/c nude mice after i.v. injected with CY7-4 (1 μmol kg^-1^) and NIR-II fluorescence guided surgery. (C) Pathologically confirmed lymph node metastases by HE section staining. Scale bar: 500 μm. **Figure S10.** Representative tumor photo of mice in each group at day 60 after treatments, n = 5. **Figure S11.** White light images of mice in fluorescence-guided surgery combined with photothermal treatment experiment. G1: partial resection + irradiation, G2: radical resection.

## Data Availability

All relevant data are available from the corresponding author upon request.
