# Supplementary material for: A mitochondrion-targeted cyanine agent for NIR-II fluorescence-guided surgery combined with intraoperative photothermal therapy to reduce prostate cancer recurrence
Source: J Nanobiotechnology. 2024 May 3;22:224. doi: 10.1186/s12951-024-02477-6 (PMC11069140; doi:10.1186/s12951-024-02477-6)
Supplement: Supplementary file 1 — Additional file 1: Figure S1. (A) Schematic illustration of CY7-4 nanocomposite preparation process. (B) The absorption spectra of CY7-4 nanocomposite in PBS with different mass ratio. (C-D) White light and NIR-II images of CY7-4 nanocomposite with different mass ratio and NIR-II fluorescence quantification. Figure S2. Hydrated particle size of CY7-4 nanocomposites (A) and pure BSA (B). (C) Zeta potential of free CY7-4. Figure S3. Physicochemical stability of free CY7-4 in DMSO and CY7-4 nanocomposites. Changes in the appearance of prepared free CY7-4 solutions (A) and CY7-4 nanocomposites (B) over a week at room temperature. Absorption spectra of prepared free CY7-4 solutions (C) and CY7-4 nanocomposites (D) at different time points. No precipitation or decrease in peak absorbance value was observed. Figure S4. (A) Photograph of NIR-II fluorescence guided PTT process. (B) Blood samples imaging with a homebuilt NIR-II imaging system. Figure S5. The IVIS imaging system acquired representative images of C57 mice at different time points (λex = 740 nm, λex = 840, auto) after i.v. injected with different SMD@BSA nanocomposite (A: CY7-1, B: CY7-2, C: CY7-3, D: CY7-4, E: ICG, 1 μmol kg-1), n = 3. Figure S6. (A-B) Representative photo of red blood cells incubated with different concentrations of CY7-4 and absorbance values at 570 nm. Figure S7. (A-C) Cell viability of L-O2, HK2, and HUVEC cells treated with different concentrations of CY7-4 for 12 or 24 hours. Figure S8. (A) The NIR-II camera acquired representative images of C57 mice at different time points (λex = 808 nm, 100 ms, 1200 nm long-pass filter) after i.v. injected with CY7-4 or ICG (1 μmol kg-1), n = 3. (B-C) Tumor fluorescence intensity and SNR quantification of CY7-4 injected mice at corresponding time points. Figure S9. (A-B) NIR-II imaging of suspicious metastases in prostate tumor bearing BALB/c nude mice after i.v. injected with CY7-4 (1 μmol kg-1) and NIR-II fluorescence guided surgery. (C) Pathological [file 12951_2024_2477_MOESM1_ESM.docx]

**A mitochondrion-targeted cyanine agent for NIR-II fluorescence-guided surgery combined with intraoperative photothermal therapy to reduce prostate cancer recurrence**

Chenchen Liu^1,2,3†^, Zong Chang^3†^, Kailei Chen^1,2†^, Qiang Xue^3^, Bingxin Shu^3^, Zhihao Wei^1,2^, Xuan Zhou^1,2^, Like Guo^3^, Yulin Zhang^3^, Yingying Pan^3^, Qi Cao^1,2^, Huageng Liang^1,2^, Qinchao Sun^3*^, Xiaoping Zhang^1,2*^

^1^Department of Urology, Union Hospital, Tongji Medical College, Huazhong University of Science and Technology, Wuhan 430022, China

^2^Institute of Urology, Union Hospital, Tongji Medical College, Huazhong University of Science and Technology, Wuhan 430022, China

^3^Guangdong Provincial Key Laboratory of Biomedical Optical Imaging Technology & Center for Biomedical Optics and Molecular Imaging, Shenzhen Institute of Advanced Technology, Chinese Academy of Science, Shenzhen 518055, China

† These authors contributed equally to this work

* Correspondence: Xiaoping Zhang, E-mail: xzhang@hust.edu.cn

Qinchao Sun, E-mail: qchao.sun@siat.ac.cn

**Experimental Section**

**Spectra measurements and photothermal properties**

The absorbance spectra (400-900 nm) of these heptamethine cyanine dyes were determined in DMSO or PBS buffer by a UV–vis scanning spectrophotometer (Shimadzu, UV-2700). Photoluminescence spectra were determined using a NIR fluorescence spectrometer (Edinburgh, FLS-920). The emission spectrum was determined with 785 nm excitation and scanning the wavelength from 800 to 1200 nm. Absolute quantum yields (QY) were measured via a quantum yield spectrometer (Hamamatsu, C11347). Briefly, the sample preparation in organic solvents involves dissolving CY7-1, CY7-2, and CY7-4 in dichloromethane, while CY7-3 and ICG were dissolved in ethanol. The sample preparation in the aqueous phase is shown in Figure S1. Adjust the absorption value of all samples to 0.1 at 785 nm. Calculate the absolute quantum yield using a blank dish without adding samples as the background.

**Dynamic light scattering measurements**

The hydrated particle size of CY7-4 nanocomposite and pure BSA was measured using a Malvern dynamic light scattering analyzer (Zetasizer Nano ZS90). The parameters were as follows: reflectance index = 1.33, absorbance = 0.01. For the zeta potentials, CY7-4 dissolved in DMSO was measured under the same equipment after proper dilution with distilled water.

**Stability of CY7-4**

The free CY7-4 and CY7-4 nanocomposites were prepared with DMSO and distilled water, respectively. The stability was determined by observing the changes of the solution appearance and absorption spectra after storing in 4 ℃ for one week.

**Tumor model**

RM-1 was cultured in a T75 flask and collected when the cell density reached 80%. The cells were resuspended with PBS and implanted subcutaneously on the back of mice (2.5×10^6^ cells). The mice were randomly divided into different groups after the tumor reached 3-5mm in diameter. Formula for calculating tumor volume: (Volume, mm^3^) = (length, mm) × (width, mm) * 2 × 1/2

***In vivo* NIR-I tumor imaging**

8 weeks C57 mice implanted with tumor were injected with different SMD (1 × 10^-6^ mol kg^-1^) intravenously (n = 3). Whole body fluorescence images at 0.5, 2, 6, 12, 24, 48 h post-injection were acquired by an IVIS imaging system (λ_ex_ = 740 nm, λ_em_ = 840 nm, exposure time: auto). The fluorescence intensity of each image was quantified via the Living Image software using ROIs to circle the region of interest.

**Cell viability**

The HUVEC, HK2, and L-O2 cells were seeded in 96-well plates (2500 cells in 150 μL medium per well). 12 hours later, the cells were cultured with different concentrations of CY7-4 for 2 hours, and then the medium was replaced with a fresh medium. After 12 or 24 hours of continuous incubation, the medium containing 10% CCK-8 (MCE) was replaced. Finally, the absorption of 450 nm was measured using a microplate reader (ThermoFisher). Cell viability was calculated by the following equation: Cell viability (%) = [A (treated)-A (blank)] / [A (no treated)-A (blank)] × 100 %. For PTT *in vitro*, cells received continuous laser irradiation (808 nm, 1 W cm^-2^) for 5 min after was CY7-4 removed. During the experiments, 500 μM N-Acetylcysteine (NAC) was added to avoid the photodynamic effect.

**Hemolysis Assay**

Fresh blood was collected from the orbital vein of male C57 mice in heparinized centrifuge tubes. After centrifugation at 5000 × g for 10 min, the supernatant was discarded, and the erythrocytes were washed thrice with saline. The concentration of erythrocytes was adjusted to 4% (w/v) with saline, and then different concentrations of CY7-4 were added. Pure saline and 0.2% (w/v) Triton X-100 were used as negative and positive controls, respectively. Absorbance values at 570 nm were determined for all samples after incubation for 4 hours at room temperature. Hemolysis rate (%) = (A _sample_ - A _negative_) / (A _positive_ - A _negative_) × 100 %.


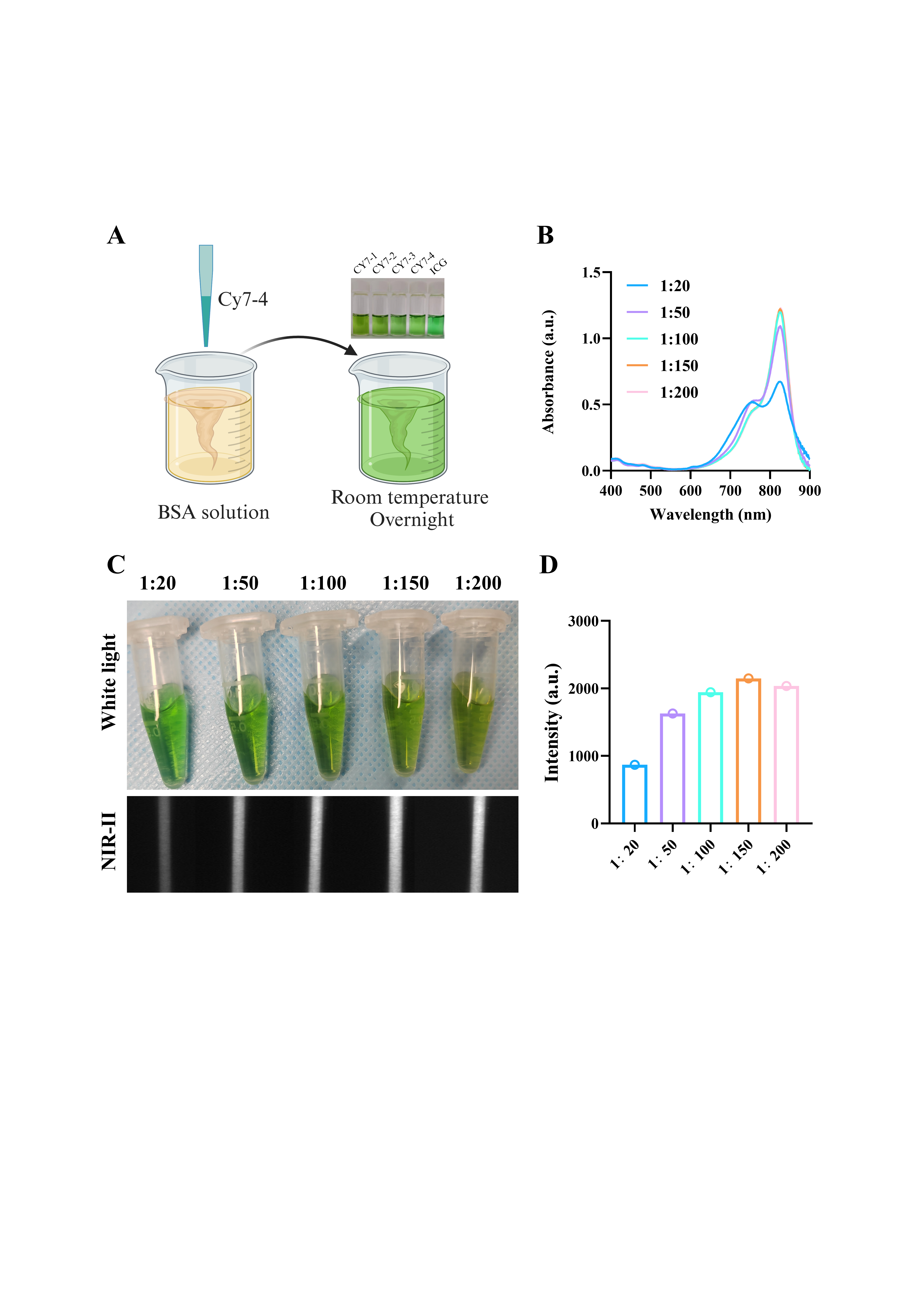


**Figure S1.** **(A)** Schematic illustration of CY7-4 nanocomposite preparation process. **(B)** The absorption spectra of CY7-4 nanocomposite in PBS with different mass ratio. **(C-D)** White light and NIR-II images of CY7-4 nanocomposite with different mass ratio and NIR-II fluorescence quantification.


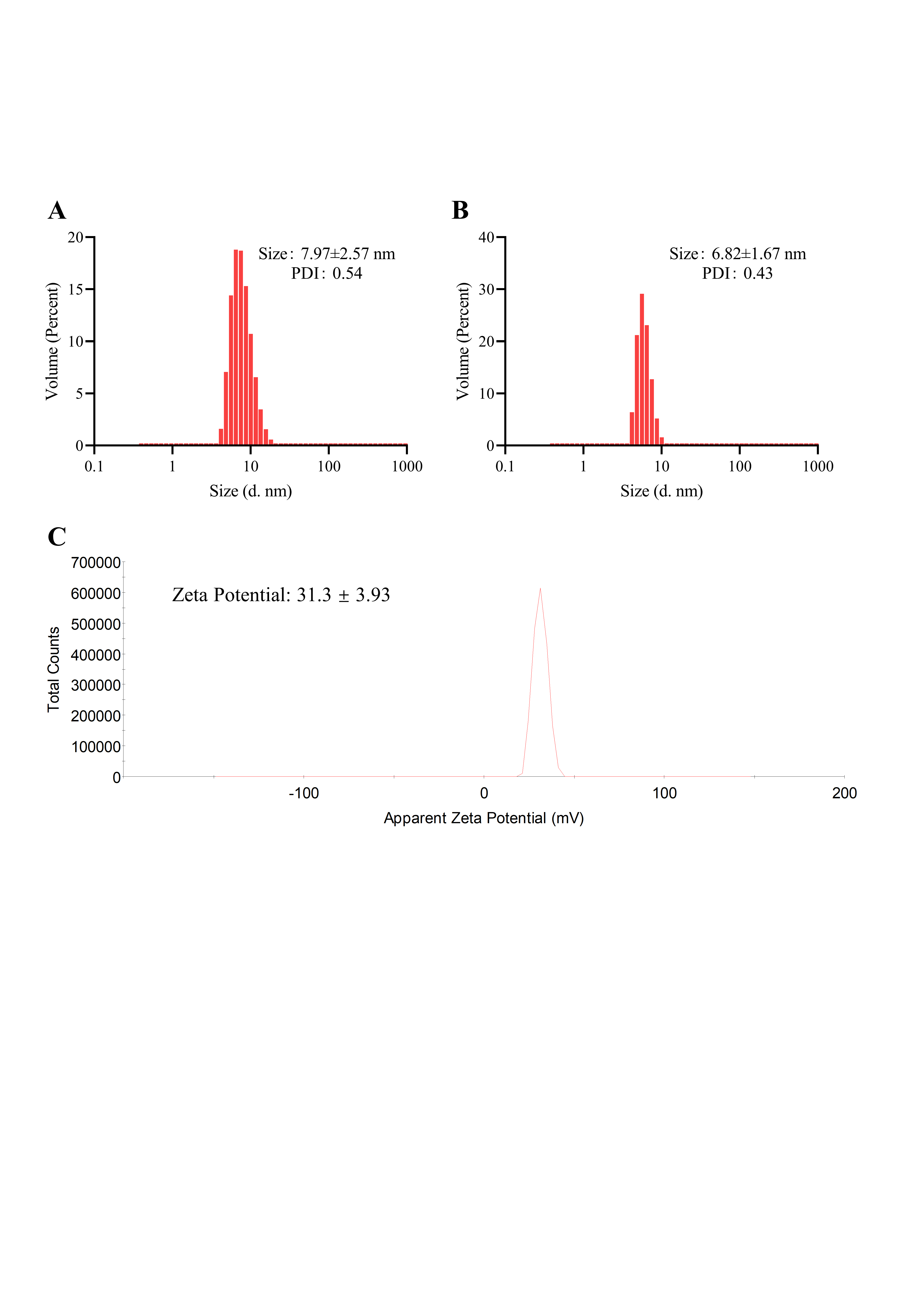


**Figure S2.** Hydrated particle size of CY7-4 nanocomposites (A) and pure BSA (B). (C) Zeta potential of free CY7-4.


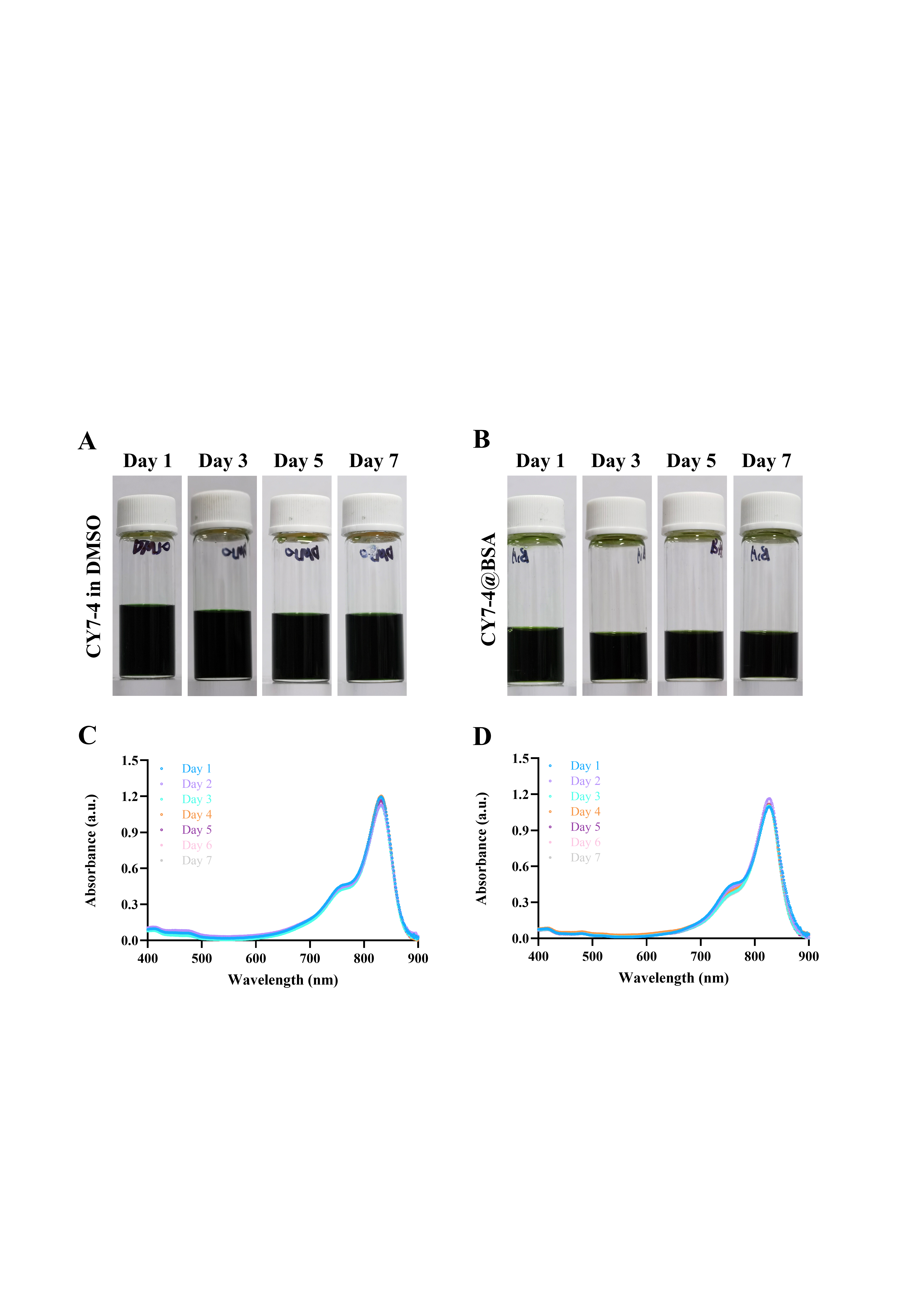


**Figure S3.** Physicochemical stability of free CY7-4 in DMSO and CY7-4 nanocomposites. Changes in the appearance of prepared free CY7-4 solutions (A) and CY7-4 nanocomposites (B) over a week at room temperature. Absorption spectra of prepared free CY7-4 solutions (C) and CY7-4 nanocomposites (D) at different time points. No precipitation or decrease in peak absorbance value was observed.


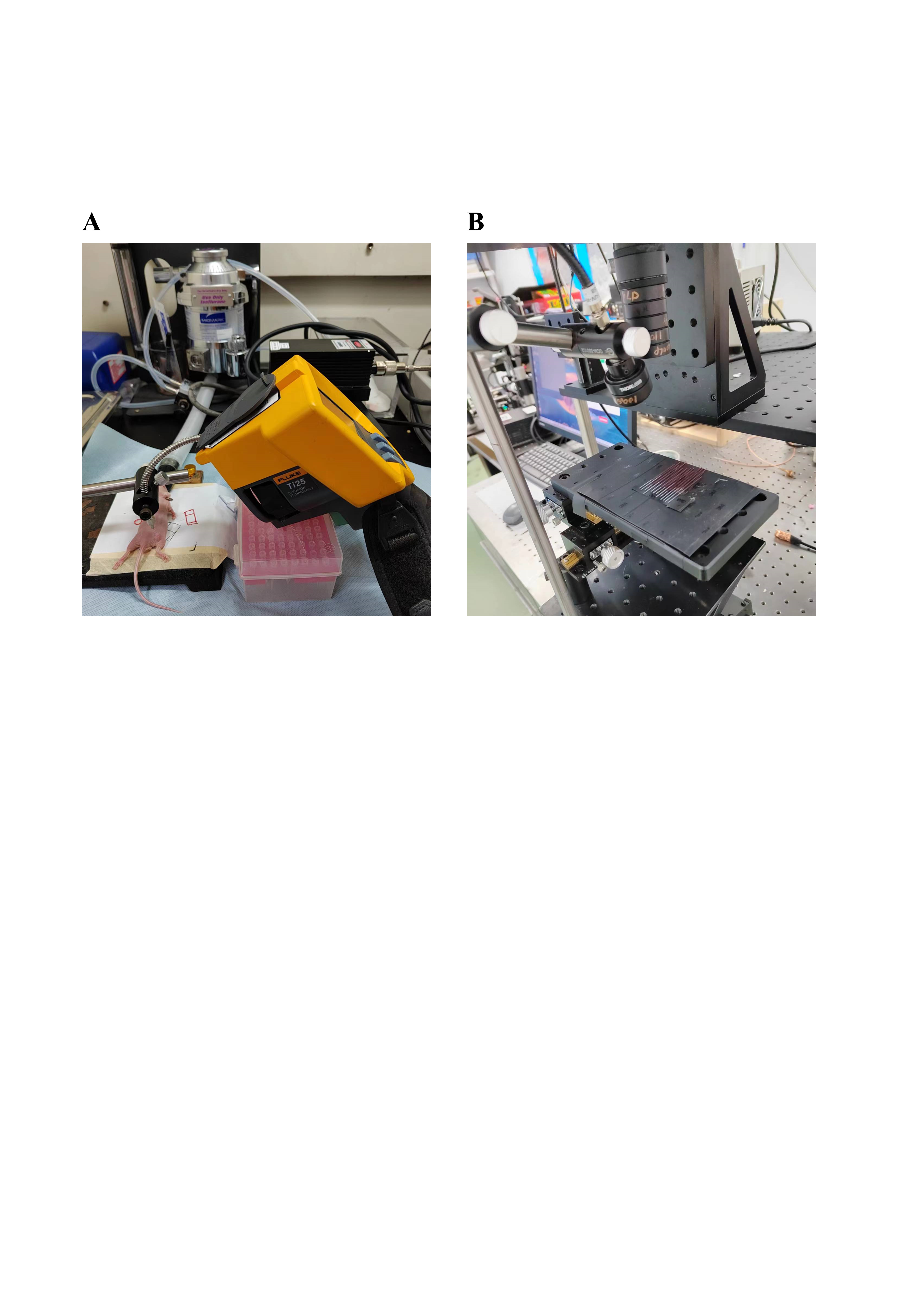


**Figure S4.** **(A)** Photograph of NIR-II fluorescence guided PTT process. **(B)** Blood samples imaging with a homebuilt NIR-II imaging system.


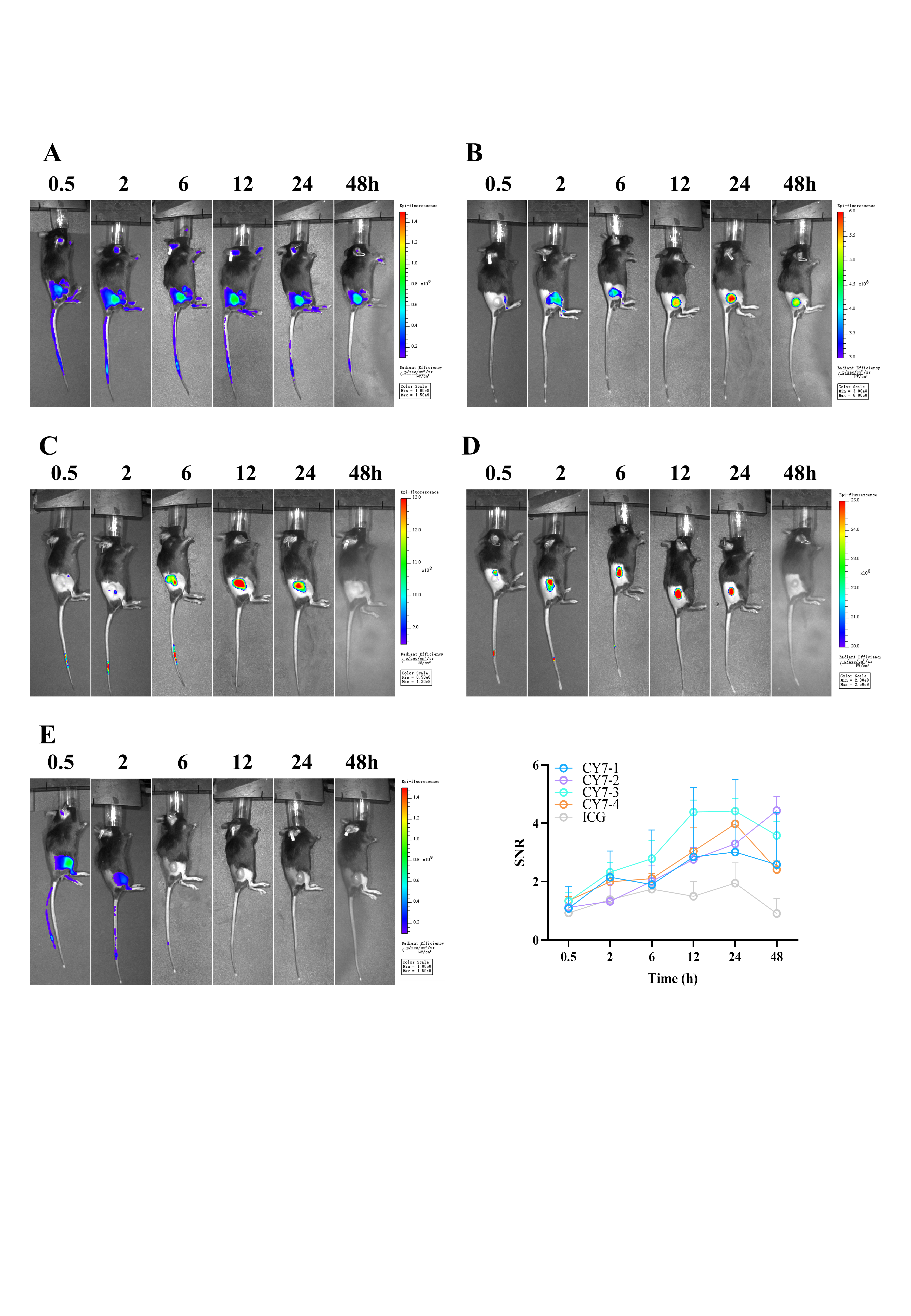


**Figure S5.** The IVIS imaging system acquired representative images of C57 mice at different time points (λ_ex_ = 740 nm, λ_ex_ = 840, auto) after i.v. injected with different SMD@BSA nanocomposite (**A**: CY7-1, **B**: CY7-2, **C**: CY7-3, **D**: CY7-4, **E**: ICG, 1 μmol kg^-1^), n = 3.


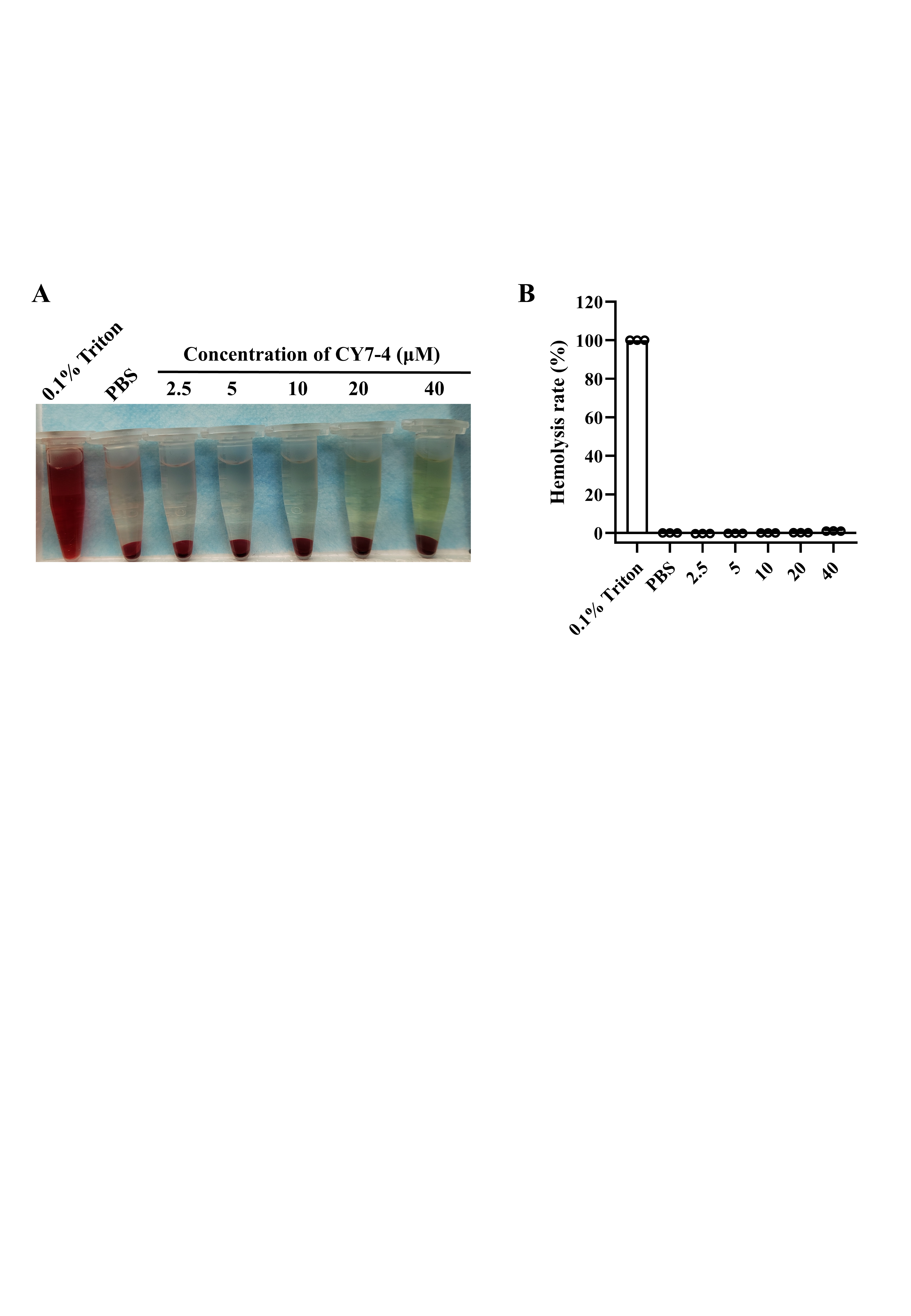


**Figure S6. (A-B)** Representative photo of red blood cells incubated with different concentrations of CY7-4 and absorbance values at 570 nm.


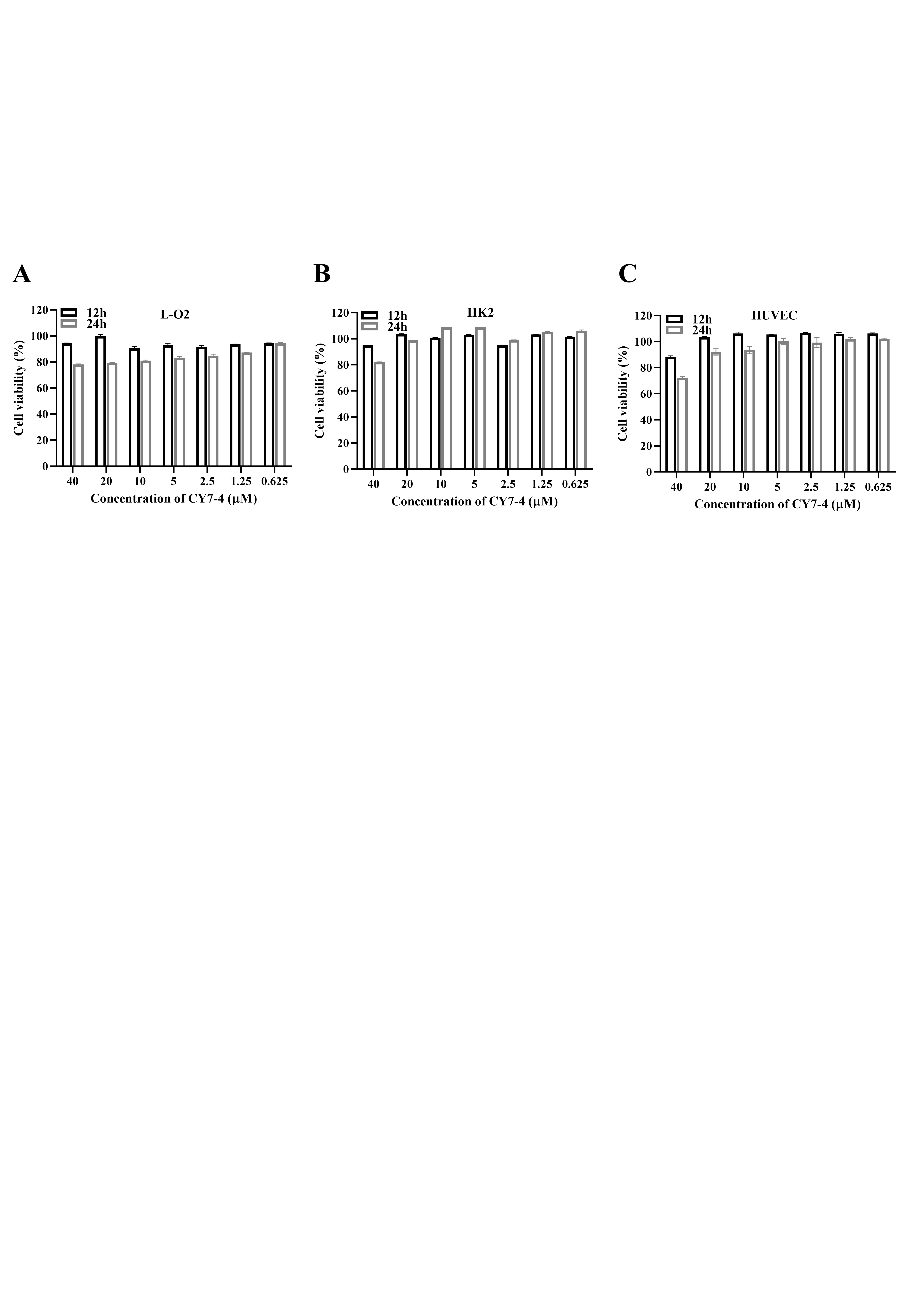


**Figure S7. (A-C)** Cell viability of L-O2, HK2, and HUVEC cells treated with different concentrations of CY7-4 for 12 or 24 hours.


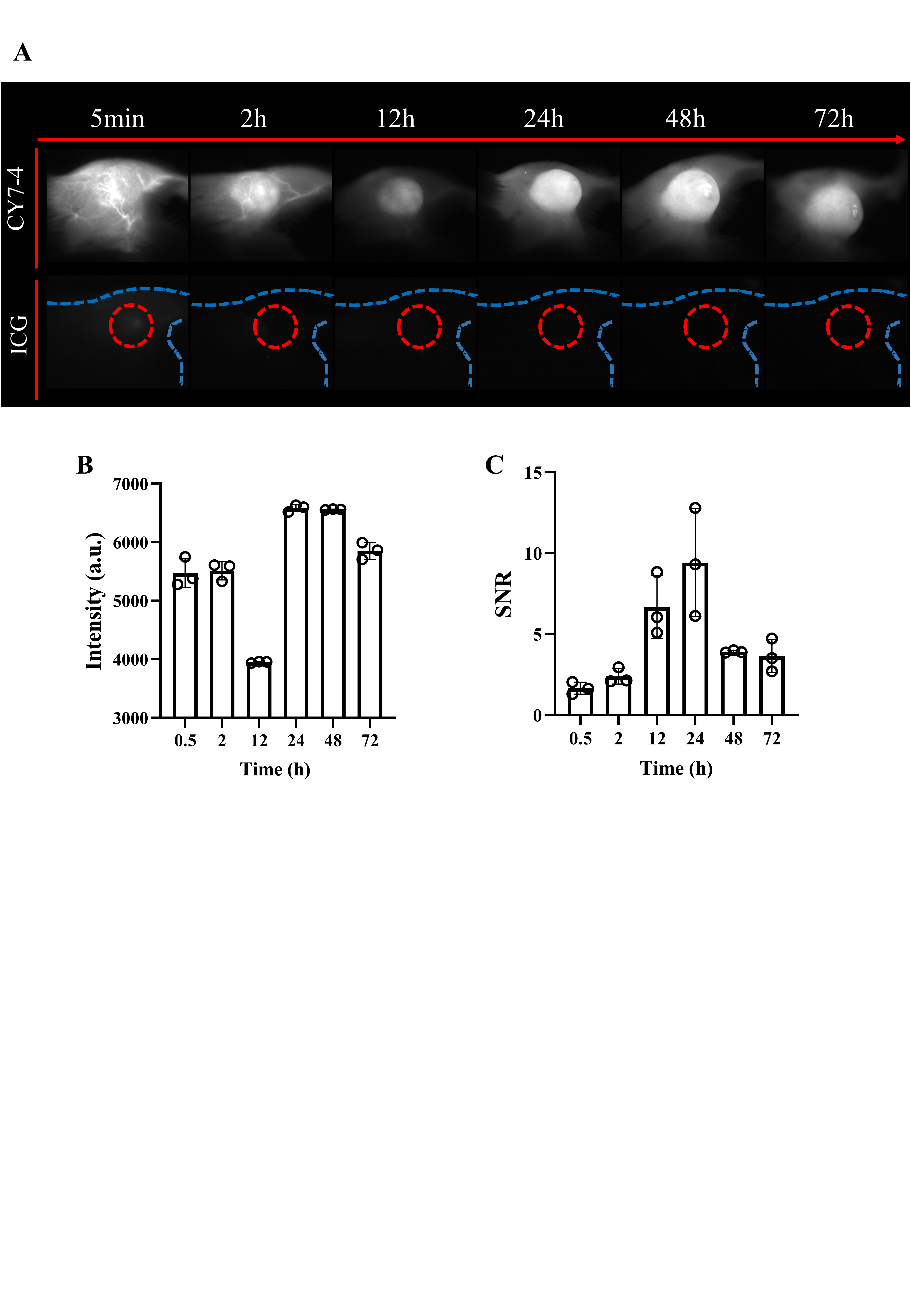


**Figure S8.** **(A)** The NIR-II camera acquired representative images of C57 mice at different time points (λ_ex_ = 808 nm, 100 ms, 1200 nm long-pass filter) after i.v. injected with CY7-4 or ICG (1 μmol kg^-1^), n = 3. **(B-C)** Tumor fluorescence intensity and SNR quantification of CY7-4 injected mice at corresponding time points.


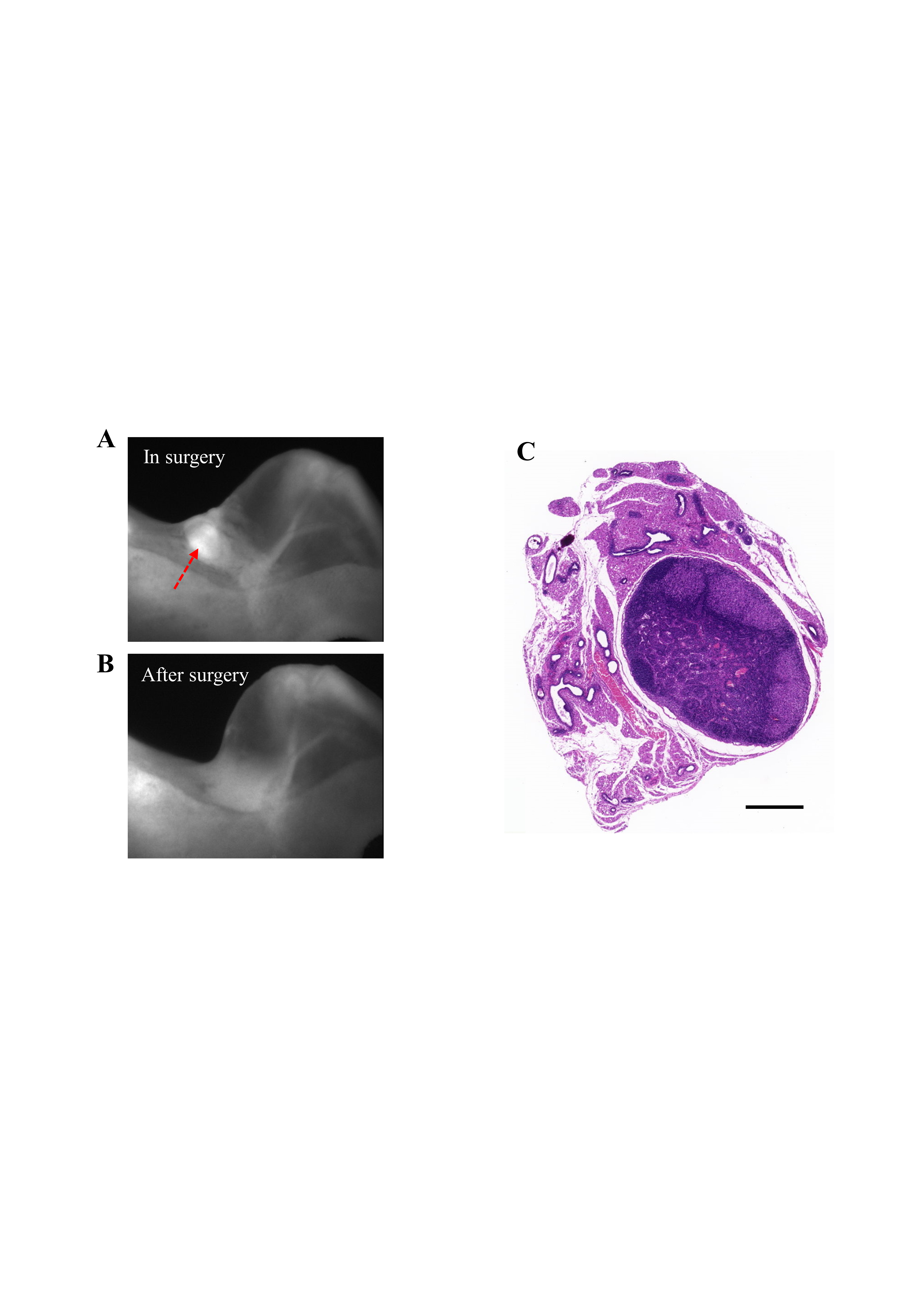


**Figure S9. (A-B)** NIR-II imaging of suspicious metastases in prostate tumor bearing BALB/c nude mice after i.v. injected with CY7-4 (1 μmol kg^-1^) and NIR-II fluorescence guided surgery. **(C)** Pathologically confirmed lymph node metastases by HE section staining. Scale bar: 500 μm.


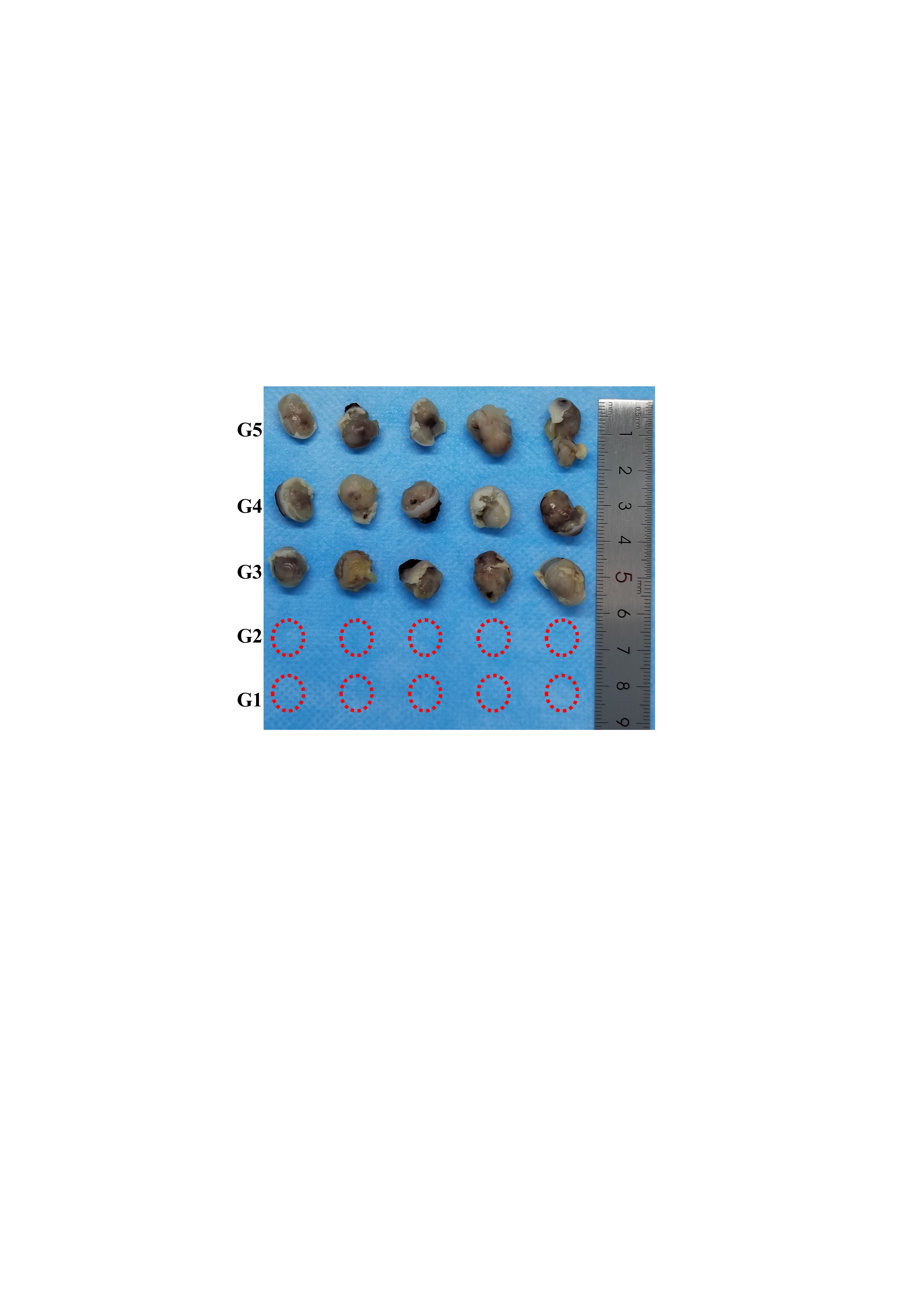


**Figure S10.** Representative tumor photo of mice in each group at day 60 after treatments, n = 5.


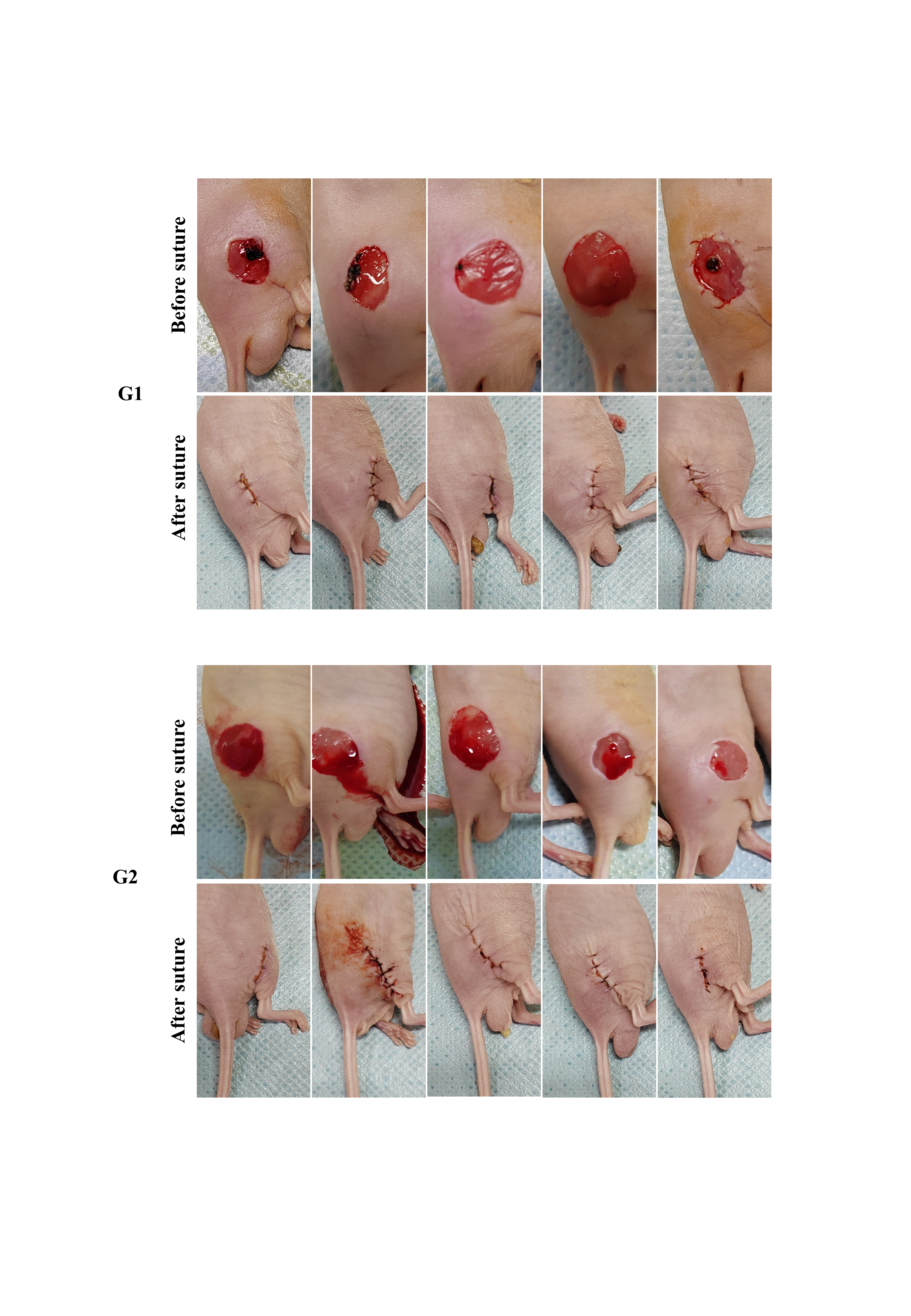


**Figure S11.** White light images of mice in fluorescence-guided surgery combined with photothermal treatment experiment. G1: partial resection + irradiation, G2: radical resection.
